# Bioactive Terphenyls Isolated from the Antarctic Lichen *Stereocaulon alpinum*

**DOI:** 10.3390/molecules27072363

**Published:** 2022-04-06

**Authors:** Kim-Hoa Phi, Min-Ji Shin, Seulah Lee, Jae Eun So, Ji Hee Kim, Sung-Suk Suh, Man Hyung Koo, Seung Chul Shin, Jin-Hyoung Kim, Jun Hyuck Lee, Ui Joung Youn

**Affiliations:** 1 Division of Life Sciences, Korea Polar Research Institute, Incheon 21990, Korea; pkh246@kopri.re.kr (K.-H.P.); cladonia@kopri.re.kr (J.E.S.); jhalgae@kopri.re.kr (J.H.K.); ssc@kopri.re.kr (S.C.S.); kimjh@kopri.re.kr (J.-H.K.); 2 Department of Polar Sciences, University of Science and Technology, Incheon 21990, Korea; junhyucklee@kopri.re.kr; 3 Department of Bioscience, Mokpo National University, Jeonnam 58554, Korea; minji4099@naver.com (M.-J.S.); sungsuksuh@mokpo.ac.kr (S.-S.S.); 4 Department of Biomedicine, Health & Life Convergence Science, BK21 Four, Mokpo National University, Jeonnam 58554, Korea; 5 Seoul School of Integrated Sciences & Technologies (aSSIST), Seoul 03767, Korea; sarahlee0801@gmail.com; 6 Research Unit of Cryogenic Novel Material, Korea Polar Research Institute, Incheon 21990, Korea; mhkoo1016@kopri.re.kr

**Keywords:** *Stereocaulon alpinum*, Antarctic lichen, terphenyl, cytotoxicity, anti-inflammation

## Abstract

Three *p*-terphenyls (**2**–**4**)—2-hydroxy-3,5-dimethoxy-*p*-terphenyl (**2**), 2-hydroxy-3,6-dimethoxy-*p*-terphenyl (**3**), and 2,3,5,6-tetramethoxy-*p*-terphenyl (**4**)—were isolated for the first time as natural products along with seven known compounds (**1**, **5**–**10**) from the Antarctic lichen *Stereocaulon alpinum.* Structures of the new compounds were elucidated by comprehensive analyses of 1D and 2D NMR and HREIMS experiments. Compound **3** exhibited cytotoxicity against HCT116 cells with the IC_50_ value of 3.76 ± 0.03 μM and also inhibited NO production in LPS-induced RAW264.7 macrophages with the IC_50_ value of 22.82 ± 0.015 μM.

## 1. Introduction

Lichens, which represent complex symbiotic associations between fungi (mycobiont) and algae (photobiont), produce various unique secondary metabolites arising from the symbiosis [1]. The two association partners play crucial roles in providing each other the conditions necessary for their continual existence [1]. Owing to this unique relationship, both mycobionts and photobionts are able to grow in environments where it is considered impossible for them to survive by themselves [1]. There are records for the use of lichens as traditional medicines by cultures in Africa, Europe, Asia, America, and Oceania [2]. People commonly use lichens to treat skin disorders, wounds, digestive and respiratory issues, obstetric, and gynecological concerns [2]. The *Stereocaulon* genus is one of the fruticose lichen groups found in a broad spectrum of regions, including tropical areas and polar zones [3]. The genus is known as the source of common depsides encountered in many lichens and it also contains unique secondary metabolites [3]. Some species of this genus are used in treating wounds, ulcers [3], urinary infection [4], and symptoms of type 2 diabetes [5]. Previous studies also reported bioactive secondary metabolites from *Stereocaulon alpinum*, including lobaric acid as an antimitotic inhibitor [6]; a cyclic depsipeptide exhibiting cytotoxicity against human tumor cell lines [7]; a depsidone with anti-proliferative activity [8]; and pseudodepsidones with antibacterial, antioxidant, and tyrosine phosphatase inhibitory activities [9,10]. However, to the best of our knowledge, there have been no reports on *Stereocaulon* genus about structural analysis and biological activities of *p*-terphenyls, which are known to restrictedly appear in fungi and lichens, and are reported to exhibit cytotoxic, antibacterial, anti-inflammatory, and antioxidant properties [11,12,13,14].

As part of our work to discover potential bioactive compounds from polar natural products, chemical analysis of the Antarctic lichen *S. alpinum* was performed and led to the isolation of four *p*-terphenyls (**1**–**4**) along with four phenolic compounds (**5**–**7**, **9**), a dibenzofuran derivative (**8**), and a steroid (**10**). Three (**2**–**4**) of the four *p*-terphenyls are reported for the first time as natural products in the current study. Herein, we describe the isolation and structural elucidation of the new compounds as well as the biological activity of all isolated compounds (**1**–**10**).

**Figure 1:**
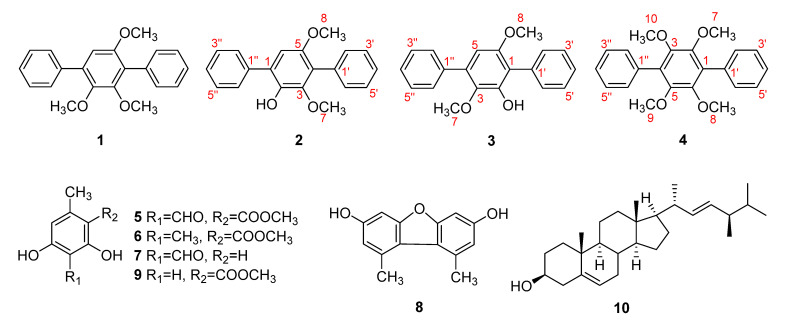
Structures of compounds **1**–**10** isolated from *S. alpinum*.

The air-dried and chopped lichen, *S. alpinum*, was extracted with methanol (MeOH) and provided the resultant MeOH extract. By using repetitive chromatographic methods, 10 compounds were isolated from the extract of *S. alpinum* (Figure 1), including three *p*-terphenyls (**2**–**4**) isolated from natural products for the first time. Compounds **2** and **3** have been reported in the process of inducing the expression of bacterial operons [15], whereas **4** was reported as a synthetic product [16]. The known compounds were identified to be 2,5,6-trimethoxy-*p*-terphenyl (**1**) [17], methyl 3-formyl-2,4-dihydroxy-6-methylbenzoate (**5**) [18], methyl 2,4-dihydroxy-3,6-dimethylbenzoate (**6**) [19], 2,6-dihydroxy-4-methylbenzaldehyde (**7**) [20], 3,7-dihydroxy-1,9-dimethyldibenzofuran (**8**) [21], methyl orsellinate (**9**) [22], and brassicasterol (**10**) [23] by analyzing their NMR spectroscopic data (see Supplementary Materials) as well as comparing them with those previously reported.

## 2. Results and Discussion

### 2.1. Structure Elucidation of the Compounds

**Figure 2:**
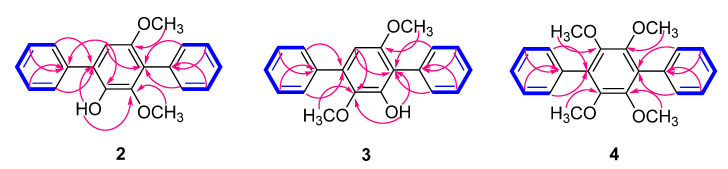
Key HMBC (pink arrows) and ^1^H-^1^H COSY (bold blue lines) correlations for compounds **2**–**4**.

Compound **2** was obtained as a yellow crystal. The molecular formula was determined as C_20_H_18_O_3_ from the [M]^+^ peak at *m*/*z* 306.1249 (calcd. for C_20_H_18_O_3_, 306.1256) in the HREIMS analysis. The ^1^H NMR data (Table 1) showed signals attributable to a hydroxy group (*δ*_H_ 5.74), 10 aromatic protons corresponding to single-substituted benzene rings (*δ*_H_ 7.37–7.67), one singlet aromatic proton at *δ*_H_ 6.75 (1H, s) and two methoxy groups at *δ*_H_ 3.37 (3H, s) and 3.72 (3H, s). The ^13^C NMR spectrum (Table 1) also showed signals of two methoxy groups (*δ*_C_ 56.6, 60.8); an upfield-shifted aromatic carbon (*δ*_C_ 108.8); four signals in the area of sp^2^ carbons (*δ*_C_ 128.2, 128.5, 129.2, 130.7), which was indicative of symmetric protonated aromatic carbons; four quaternary aromatic carbons (*δ*_C_ 123.3, 126.9, 133.5, 137.9); and three oxygenated aromatic carbons (*δ*_C_ 140.6, 145.5, 150.4). The NMR data of **2** were very similar to those of **1** [15], 2,5,6-trimethoxy-*p*-terphenyl, where the only difference appeared to be the existence of a hydroxy group instead of a methoxy group (Figure 1). Based on ^1^H and ^13^C NMR data combined with 2D NMR spectra (see Supplementary Materials), compound **2** appeared to possess a *p*-terphenyl skeleton as in **1**, bearing three oxygenated functionalities, the benzene ring at the center is substituted by a hydroxy and two methoxy groups. Positions of the three substituents were assigned by HMBC correlations from H-6 to C-4 (*δ*_C_ 123.3) and C-2 (*δ*_C_ 140.6), from H_3_-8 to C-5 (*δ*_C_ 150.4), from H_3_-7 to C-3 (*δ*_C_ 145.5), and from 2-OH to C-1 (*δ*_C_ 126.9) and C-3 (Figure 2). HMBC correlations from H-2′/H-6′ to C-4, H-2″/H-6″ to C-1 as well as from H-6 to C-1″ (*δ*_C_ 137.9) confirmed linkages between the benzene rings, and cross-peaks between H-2′/H-3′/H-4′/H-5′/H-6′ and H-2″/H-3″/H-4″/H-5″/H-6″ observed in ^1^H-^1^H COSY spectrum further supported the existence of the two single-substituted benzene rings. An existence of the unusual correlation between H_3_-8 (*δ*_H_ 3.72, s) and H-6 (*δ*_H_ 6.75, s) was recorded in the COSY spectrum. This phenomenon happened because the aromatic system of compound **2** allows observing “through-space” couplings between methyl and hydrogen which were in close proximity [24,25]. In this case, it is a long-range coupling (five bonds) between hydrogen from the methoxy group attached to an aromatic ring and the ortho-positioned hydrogen atom, which was also reported in previous studies [26]. Combining the above, the complete structure of **2** was established as a new compound 2-hydroxy-3,5-dimethoxy-p-terphenyl (Figure 1).

Compound **3** was obtained as a yellow crystal, and the molecular formula was established as C_20_H_18_O_3_ from the peak [M]^+^ at *m*/*z* 306.1253 (calcd. for C_20_H_18_O_3,_ 306.1256) observed in the HREIMS analysis. The ^1^H and ^13^C NMR data (Table 1) supported that the structure of **3** was very similar to that of **2**. The only difference appeared to be the arrangement of the functional groups attached to the central benzene ring. The ^1^H NMR spectrum of **3** showed proton signals corresponding to two methoxy groups [(*δ*_H_ 3.75 (3H, s), 3.45 (3H, s)], a hydroxy group [*δ*_H_ 5.95 (1H, s)] and eleven aromatic protons [(*δ*_H_ 6.51 (1H, s), 7.38 (2H, m), 7.47 (6H, m), 7.66 (2H, m)]. HMBC correlations from H_3_-8 to C-6 (*δ*_C_ 153.6), H_3_-7 to C-3 (*δ*_C_ 139.0), 2-OH to C-1 (*δ*_C_ 117.1), and C-3 confirmed the positions of substituents on the center benzene ring, placing a methoxy group at C-3 and a hydroxy group at C-2, and correlations from H-2′/H-6′ to C-1 and from H-2″/H-6″ to C-4 (*δ*_C_ 133.1) further supported the connection between the rings (Figure 2). As in compound **2**, cross-peaks between H-2′/H-3′/H-4′/H-5′/H-6′ and H-2″/H-3″/H-4″/H-5″/H-6″ were observed in the ^1^H-^1^H COSY spectrum. Based on the above data, compound **3** was determined to be 2-hydroxy-3,6-dimethoxy-p-terphenyl (Figure 1).

Compound **4** was obtained as a colorless crystal, and the HREIMS data of **4** provided the [M]^+^ peak at *m*/*z* 350.1521 (calcd. for C_22_H_22_O_4,_ 350.1518), indicating the molecular formula to be C_22_H_22_O_4_. This compound was also assumed to share the same skeleton with compounds **2** and **3**, with four methoxy groups attached to the central benzene ring. Based on the ^1^H and ^13^C NMR data (Table 1), compound **4** seemed to be symmetrical, where proton signals were observed at *δ*_H_ 3.59 (12H, s), 7.38 (2H, m), and 7.44 (8H, m), and only seven peaks accounting for 22 carbons appeared in the ^13^C spectrum (*δ*_C_ 61.0, 127.3, 128.0, 130.3, 130.4, 134.0, 147.2) (Figure S17). Positions of the methoxy groups to the central benzene ring were confirmed by HMBC correlations from H_3_-7/8/9/10 (*δ*_H_ 3.59) to C-2/3/5/6 (*δ*_C_ 147.2), and the connection between the rest benzene rings was supported by HMBC correlations from H-2′/H-6′ to C-1 (*δ*_C_ 130.4) and from H-2″/H-6″ to C-4 (*δ*_C_ 130.4) (Figure 2). Accordingly, the complete structure of **4** was elucidated as 2,3,5,6-tetramethoxy-*p*-terphenyl (Figure 1).

### 2.2. Biological Evaluation

All isolated compounds (**1**–**10**) were evaluated for cytotoxicity and anti-inflammatory activity. Compound **3** exhibited significant cytotoxicity against HCT116 cells, human colorectal carcinoma cell line, with the IC_50_ value of 3.76 ± 0.03 μM (Table S1), and it also displayed inhibitory activity on NO production in LPS-induced RAW264.7 macrophages, in which the IC_50_ value was 22.82 ± 0.015 μM (Table S2). Four compounds (**1**–**4**) possess similar structures, but only compound **3**—which has a hydroxy group attached to C-5 and a methoxy group attached to C-6 of the central benzene ring—showed its activities through the assays. Consequently, although the structure–activity relationships of the *p*-terphenyls have not been investigated thoroughly, our results suggested the possibility of these functional groups and their positions to be responsible for the biological activities of *p*-terphenyls.

## 3. Materials and Methods

### 3.1. General Procedures

Mass spectra were recorded on JEOL JMS-700 mass spectrometer (JEOL Ltd., Tokyo, Japan). NMR spectra were acquired on a Bruker AVANCE III operating at 600 MHz (^1^H) and 150 MHz (^13^C) (Bruker, MA, USA). Semi-preparative HPLC was performed on YL9100 HPLC system (Young Lin, Anyang, Korea) equipped with a PDA detector (200–600 nm) using a normal-phase YMC-Pack SIL-HG column (250 × 20 mm I.D., 10 μm) and a reserved-phase YMC-Pack Pro C18 column (250 × 20 mm I.D., 10 μm). Column chromatography (CC) was conducted on silica gel (0.063–0.200 mm, Merck, Darmstadt, Germany) and RP-18 (YMC·GEL ODS-A, 12 nm, S-150 μm). Normal-phase thin-layer chromatography (TLC) was carried out on glass plates pre-coated with silica gel 60 F_254_ (0.25 mm, Merck, Darmstadt, Germany), mobile phase hexane:EtOAc (9:1 to 4:6); CHCl_3_:MeOH (19:1 to 1:1). Reversed-phase TLC was carried out on glass plates pre-coated with RP-18 F_254_ (0.25 mm, Merck, Darmstadt, Germany), mobile phase MeOH:H_2_O (50:50 to 100:0), and MeCN:H_2_O (50:50 to 100:0). Spots on TLC plates were detected using UV lamp (254 nm and 365 nm), and heating after dipping in 20% sulfuric acid in H_2_O. The sample was injected into the semi-prep. HPLC was carried out by multiple injections with the concentration of each single injection being around 10 mg/1 mL and the injected volume being approximately 200 μL.

### 3.2. Lichen Material

The lichen was collected from King George Island, Antarctica, and identified by Dr. Ji Hee Kim. A voucher specimen was deposited at the Polar Natural Product Chemistry Laboratory of the Korea Polar Research Institute.

### 3.3. Extraction and Isolation

The air-dried and chopped lichen (300 g) was macerated with methanol (3 L × 3 times) at room temperature for three days. The crude extract was concentrated under a vacuum to yield a brown slurry (22 g). The methanol extract was then suspended in H_2_O and sequentially partitioned with *n*-hexane, ethyl acetate (EtOAc), and *n*-buthanol (BuOH) to afford the hexane (1.05 g), EtOAc (3.44 g), and BuOH (1.91 g) soluble fractions. The hexane fraction (1.05 g) was adsorbed onto a silica gel column chromatography (CC), and gravity elution was performed with the gradient solvent system of hexane with increasing amounts of EtOAc (5–100%), followed by mixture of chloroform (CHCl_3_) and MeOH (from 0% to 50% MeOH in CHCl_3_) to yield 15 subfractions (H1–H15). H1 (447.0 mg) was separated into four fractions (H1.1–H1.4) using silica gel CC with a gradient solvent system of hexane:EtOAc (95:5–50:50). Fraction H1.1 (119.0 mg) was purified by HPLC eluted with hexane:EtOAc (95:05, 2 mL/min) using a normal-phase YMC-Pack SIL-HG column (250 × 20 mm I.D., 10 μm) to obtain subfraction H1.1.1 and **4** (*t_R_* 64.0 min, 4.0 mg). Subfraction H1.1.1 (58.0 mg) was loaded and eluted with 100% MeOH through a Sep-Pak Cartridge packed with C-18 particles to give **1** (22.0 mg). Fraction H1.3 (57.0 mg) was subjected to HPLC and eluted with hexane:EtOAc (95:05, 2 mL/min) using a normal-phase YMC-Pack SIL-HG column (250 × 20 mm I.D., 10 μm) to afford four subfractions (H1.3.1–H1.3.4). Subfraction H1.3.3 (13.0 mg) was further purified on HPLC and eluted with MeOH:H_2_O (90:10, 3 mL/min) using a reserved-phase YMC-Pack Pro C18 column (250 × 20 mm I.D., 10 μm) to yield **2** (*t_R_* 29.3 min, 8.0 mg) and **5** (*t_R_* 33.8 min, 2.0 mg). Isolation of H1.3.4 using HPLC (MeOH:H_2_O, 90:10, 3 mL/min) with a reserved-phase YMC-Pack Pro C18 column (250 × 20 mm I.D., 10 μm) gave compound **3** (*t_R_* 27.2 min, 5.0 mg). Compound **6** *(t_R_* 53.0 min, 5.0 mg) was obtained from fraction H3 (107.0 mg) using a C-18 CC (MeCN:H_2_O, 50:50 to 80:20) followed by HPLC purification with hexane:EtOAc (80:20, 2 mL/min) using a normal-phase YMC-Pack SIL-HG column (250 × 20 mm I.D., 10 μm). Fraction H6 (36.0 mg) was subjected to HPLC (hexane:EtOAc, 80:20, 2 mL/min) using a normal-phase YMC-Pack SIL-HG column (250 × 20 mm I.D., 10 μm) and compound **9** (*t_R_* 59.9 min, 1.0 mg) was obtained.

The EtOAc fraction (3.44 g) was subjected to silica gel CC, and gravity elution was performed with a gradient solvent system of hexane with increasing amounts of EtOAc (5–100%), followed by CHCl_3_ and MeOH (from 0% to 50% MeOH) to afford eight fractions (E1–E8). Purification of E2 (46.0 mg) was conducted by HPLC (MeOH:H_2_O, 80:20, 2 mL/min) using a reserved-phase YMC-Pack Pro C18 column (250 × 20 mm I.D., 10 μm), which resulted in the isolation of additional amounts of compound **5** (*t_R_* 76.7 min, 7.0 mg). During the process of eluting E3 (116.0 mg) using C-18 CC with gradient solvent system of MeOH:H_2_O (50:50–80:20), E3.3 (48.0 mg) was collected and was further isolated. Compound **10** (5.0 mg) was obtained by purifying a portion of E3.3 using HPLC (hexane:EtOAc 70:30, 3 mL/min) equipped with a normal-phase YMC-Pack SIL-HG column (250 × 20 mm I.D., 10 μm), and the remaining portion of E3.3 was subjected to normal-phase preparative TLC plates and eluted with 100% dichloromethane and then purified with HPLC (MeOH:H_2_O, 80:20, 3 mL/min) equipped with a reserved-phase YMC-Pack Pro C18 column (250 × 20 mm I.D., 10 μm) to give additional amounts of compound **6** (*t_R_* 31.9 min, 5.0 mg). Subfraction E4 (27.0 mg) was isolated by HPLC with a solvent system of MeOH:H_2_O (86:14, 2 mL/min) using a reserved-phase YMC-Pack Pro C18 column (250 × 20 mm I.D., 10 μm) to yield **7** (*t_R_* 35.0 min, 7.0 mg). Compound **8** (*t_R_* 47.9 min, 1.0 mg) was isolated from fraction E5 (22.0 mg) by HPLC (MeOH:H_2_O, 72:28–93:07, 2mL/min) with a reserved-phase YMC-Pack Pro C18 column (250 × 20 mm I.D., 10 μm).

2,6-Dimethoxy-5-hydroxy-*p*-terphenyl (**2**): Yellow crystal; (+)-HREIMS *m*/*z* 306.1249 [M]+ (calcd. for C_20_H_18_O_3_, 306.1256); for ^1^H and ^13^C NMR spectroscopic data, see Table 1.

2,5-Dimethoxy-6-hydroxy-*p*-terphenyl (**3**): Yellow crystal; (+)-HREIMS *m*/*z* 306.1253 [M]^+^ (calcd. for C_20_H_18_O_3,_ 306.1256); for ^1^H and ^13^C NMR spectroscopic data, see Table 1.

2,3,5,6-Tetramethoxy-*p*-terphenyl (**4**): Colorless crystal; (+)-HREIMS *m*/*z* 350.1521 [M]^+^ (calcd. for C_22_H_22_O_4,_ 350.1518); for ^1^H and ^13^C NMR spectroscopic data, see Table 1.

### 3.4. Cell Culture

RAW264.7 macrophages and HCT116 were cultured in Dulbecco’s modified Eagle’s medium (DMEM) supplemented with 10% fetal bovine serum (FBS) and 1% penicillin at 37 °C in a humidified CO_2_ incubator. In this study, macrophages were subjected in the absence or presence of the isolated compounds with different concentrations. Isolated compounds were added 1 h prior to LPS (0.5 μg/mL) stimulation. HCT116 cells were seeded 5 × 10^3^ cells/well on a 96-well plate in triplicate, and then incubated in 5% CO_2_ supplement at 37 °C.

### 3.5. MTS Assay

MTS assay was conducted to determine the cytotoxic effects of the isolated compounds against HCT116 cells. The cells were seeded at a density of 2 × 10^5^ cells/mL on a 96-well plate. After incubating in 24 h, 10% MTS solution was added to the cell culture medium, and then it was further incubated at 37 °C for 1 h. The concentration of the treated compounds ranged from 50 μM to 6.25 μM, using serial dilution. The absorbance was measured after 24 h using a microplate reader (Promega, Madison, WI, USA) at 490 nm.

### 3.6. Measurement of Nitric Oxide (NO) Production

NO concentration in the RAW264.7 cell culture supernatant was measured using Griess reagent. Briefly, 100 μL of the collected supernatant was mixed with equal amounts of Griess reagent (1% sulfanilamide in 5% phosphoric acid, 0.1% *N*-(1-naphthyl) ethylenediamine). The mixtures were incubated for 10 min at room temperature, and then the absorbance value of each well was determined at a wavelength of 540 nm using a microplate reader. Nitrite concentration was determined using a sodium nitrite calibration curve (0–100 μM).

## Figures and Tables

**Table 1:** ^1^H (600 MHz) and ^13^C NMR (150 MHz) data of compounds **2**-**4** in CDCl_3_.

No.	2		3		4	
	*δ*_H_ (*J* in Hz)	*δ*_C_	*δ*_H_ (*J* in Hz)	*δ*_C_	*δ*_H_ (*J* in Hz)	*δ*_C_
1		126.9		117.1		130.3
2		140.6		147.3		147.2
3		145.5		139.0		147.2
4		123.3		133.1		130.3
5		150.4	6.51, s	104.1		147.2
6	6.75, s	108.8		153.6		147.2
7	3.37, s	60.8	3.45, s	61.1	3.59, s	61.0
8	3.72, s	56.6	3.75, s	56.1	3.59, s	61.0
9					3.59, s	61.0
10					3.59, s	61.0
1′		133.5		133.3		134.0
2′/6′	7.49, m	128.2	7.47, m	130.9	7.44, m	130.4
3′/5′	7.45, m	130.7	7.47 m	128.2	7.44, m	128.0
4′	7.37, td (7.5, 1.2)	127.5	7.38, m	127.5	7.38, m	127.3
1″		137.9		138.2		134.0
2″/6″	7.67, dd (8.3, 1.2)	128.5	7.66, dd (8.3, 1.3)	128.9	7.44, m	130.4
3″/5″	7.47	129.2	7.47, m	128.7	7.44, m	128.0
4″	7.37, td (7.5, 1.2)	127.5	7.38 m	127.7	7.38, m	127.3
2-OH	5.74, s		5.95, s			

Assignments were determined by HSQC, HMBC, and H-H COSY experiments. Coupling constants (in Hz) are in parentheses.

## Data Availability

Not applicable.

## Supplementary Materials

The following supporting information can be downloaded at: https://www.mdpi.com/article/10.3390/molecules27072363/s1, Figure S1: ^1^H NMR spectrum of compound **1** in chloroform-*d* (600 MHz); Figure S2: ^13^C NMR spectrum of compound **1** in chloroform-*d* (150 MHz); Figure S3: HR-EIMS positive spectrum of compound **2**; Figure S4: ^1^H NMR spectrum of compound **2** in chloroform-*d* (600 MHz); Figure S5: ^13^C NMR spectrum of compound **2** in chloroform-*d* (150 MHz); Figure S6: HSQC NMR spectrum of compound **2** in chloroform-*d* (600 MHz); Figure S7: COSY NMR spectrum of compound **2** in chloroform-*d* (600 MHz); Figure S8: HMBC NMR spectrum of compound **2** in chloroform-*d* (600 MHz); Figure S9: HR-EIMS positive spectrum of compound 3; Figure S10: ^1^H NMR spectrum of compound 3 in chloroform-*d* (600 MHz); Figure S11: ^13^C NMR spectrum of compound **3** in chloroform-*d* (150 MHz); Figure S12: HSQC NMR spectrum of compound **3** in chloroform-*d* (600 MHz); Figure S13: COSY NMR spectrum of compound **3** in chloroform-*d* (600 MHz); Figure S14: HMBC NMR spectrum of compound **3** in chloroform-*d* (600 MHz); Figure S15: HR-EIMS positive spectrum of compound **4**; Figure S16: ^1^H NMR spectrum of compound **4** in chloroform-*d* (600 MHz); Figure S17: ^13^C NMR spectrum of compound 4 in chloroform-*d* (150 MHz); Figure S18: HSQC NMR spectrum of compound **4** in chloroform-*d* (600 MHz); Figure S19: COSY NMR spectrum of compound **4** in chloroform-*d* (600 MHz); Figure S20: HMBC NMR spectrum of compound **4** in chloroform-*d* (600 MHz); Figure S21: ^1^H NMR spectrum of compound **5** in chloroform-*d* (600 MHz); Figure S22: ^1^H NMR spectrum of compound 6 in chloroform-*d* (600 MHz); Figure S23: ^1^H NMR spectrum of compound 7 in DMSO-*d6* (600 MHz); Figure S24: ^1^H NMR spectrum of compound **8** in methanol-*d4* (600 MHz); Figure S25: ^1^H NMR spectrum of compound **9** in chloroform-*d* (600 MHz); Figure S26: ^1^H NMR spectrum of compound **10** in chloroform-*d* (600 MHz); Table S1: Cytotoxicity of compounds **3** against HCT116 cells; Table S2: NO inhibition of compounds **3** against LPS-induced RAW264.7 cells.
