# Supplementary material for: Bioactive Terphenyls Isolated from the Antarctic Lichen Stereocaulon alpinum
Source: Molecules. 2022 Apr 6;27(7):2363. doi: 10.3390/molecules27072363 (PMC9000585; doi:10.3390/molecules27072363)
Supplement: Supplementary file 1 [file molecules-27-02363-s001.zip › molecules-1661273-supplementary.pdf]

Bioactive Terphenyls Isolated from the Antarctic lichen *Stereocaulon alpinum*

Kim-Hoa Phi [a,b],Min-Ji Shin,[c,d] Seulah Lee [e], Jae Eun So [a,b], Ji Hee Kim [a], Sung-Suk Suh [c], Man Hyung Koo [f], Seung Chul Shin,[a] Jin-Hyoung Kim,[a,b] Jun Hyuck Lee [b,f] and Ui Joung Youn [a,b,*]

[a] Division of Life Sciences, Korea Polar Research Institute, Incheon 21990, Republic of Korea

[b] Department of Polar Sciences, University of Science and Technology, Incheon 21990, Republic of Korea

[c] Department of Bioscience, Mokpo National University, Joennam 58554, Republic of Korea

[d] Department of Biomedicine, Health & Life Convergence Science, BK21 Four, Mokpo National University, Joennam 58554, Republic of Korea

[e] Seoul School of Integrated Sciences & Technologies (aSSIST), Seoul 03767, Republic of Korea

[f] Research Unit of Cryogenic Novel Material, Korea Polar Research Institute, Incheon 21990, Republic of Korea

*****Correspondence: ujyoun@kopri.re.kr; Tel.: +82-32-760-5562

Correspondence to:

TEL: +82-32-760-5562; FAX: +82-32-760-5509; E-mail: ujyoun@kopri.re.kr

Table of Contents

Figure S1. ^{1}H NMR spectrum of compound **1** in chloroform-*d* (600 MHz) ……………………………. **3**

Figure S2. ^{13}C NMR spectrum of compound **1** in chloroform-*d* (150 MHz) …………………………… **4**

Figure S3. HR-EIMS positive spectrum of compound **2**…………………………………………………… **5**

Figure S4. ^{1}H NMR spectrum of compound **2** in chloroform-*d* (600 MHz) …………………………… **6**

Figure S5. ^{13}C NMR spectrum of compound **2** in chloroform-*d* (150 MHz) ……………………………. **7**

Figure S6. HSQC NMR spectrum of compound **2** in chloroform-*d* (600 MHz) ………………………… **8**

Figure S7. COSY NMR spectrum of compound **2** in chloroform-*d* (600 MHz) ………………………… **9**

Figure S8. HMBC NMR spectrum of compound **2** in chloroform-*d* (600 MHz) ……………………… **10**

Figure S9. HR-EIMS positive spectrum of compound **3**…………………………………………………... **11**

Figure S10. ^{1}H NMR spectrum of compound **3** in chloroform-*d* (600 MHz) ..…………………………… **12**

Figure S11. ^{13}C NMR spectrum of compound **3** in chloroform-*d* (150 MHz)..………………………… **13**

Figure S12. HSQC NMR spectrum of compound **3** in chloroform-*d* (600 MHz) ……………………… **14**

Figure S13. COSY NMR spectrum of compound **3** in chloroform-*d* (600 MHz) ……………………… **15**

Figure S14. HMBC NMR spectrum of compound **3** in chloroform-*d* (600 MHz) ……………………... **16**

Figure S15. HR-EIMS positive spectrum of compound **4**………………………………………………. **17**

Figure S16. ^{1}H NMR spectrum of compound **4** in chloroform-*d* (600 MHz) ………………………….. **18**

Figure S17. ^{13}C NMR spectrum of compound **4** in chloroform-*d* (150 MHz)………………………….. **19**

Figure S18. HSQC NMR spectrum of compound **4** in chloroform-*d* (600 MHz) ……………………… **20**

Figure S19. COSY NMR spectrum of compound **4** in chloroform-*d* (600 MHz) ……………………… **21**

Figure S20. HMBC NMR spectrum of compound **4** in chloroform-*d* (600 MHz) ……………………... **22**

Figure S21. ^{1}H NMR spectrum of compound **5** in chloroform-*d* (600 MHz)…………………………... **23**

Figure S22. ^{1}H NMR spectrum of compound **6** in chloroform-*d* (600 MHz)…………………………... **24**

Figure S23. ^{1}H NMR spectrum of compound **7** in DMSO-*d6* (600 MHz)……………………………… **25**

Figure S24. ^{1}H NMR spectrum of compound **8** in methanol-*d4* (600 MHz)…………………….…... **26**

Figure S25. ^{1}H NMR spectrum of compound **9** in chloroform-*d* (600 MHz)…………………..……. **27**

Figure S26. ^{1}H NMR spectrum of compound **10** in chloroform-*d* (600 MHz)……………………….... **28**

Table S1. Cytotoxicity of compounds **3** against HCT116 cells……………………………………… **29**

Table S2. NO inhibition of compounds **3** against LPS-induced RAW264.7 cells……………………**29**

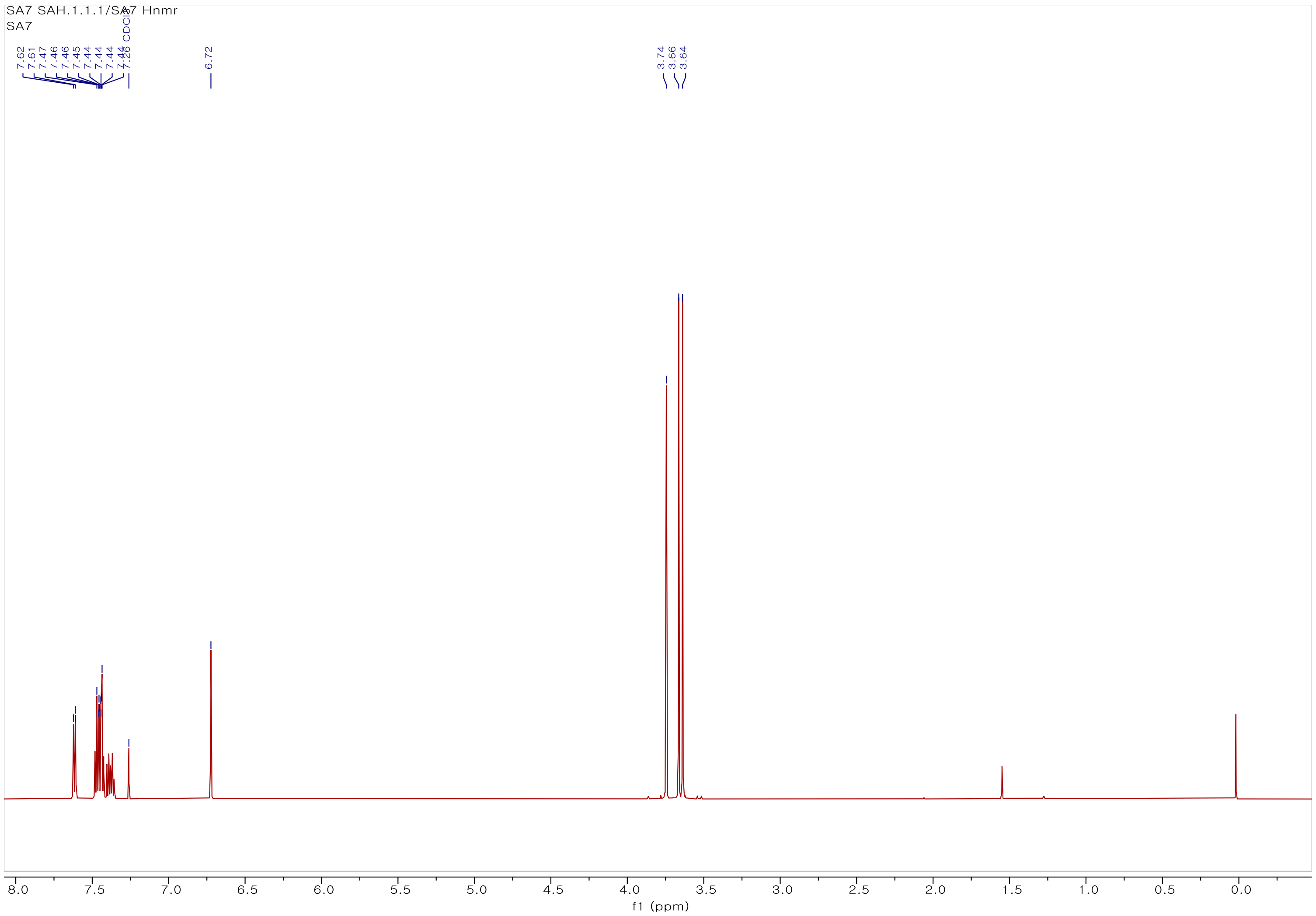

Figure S1. ^{1}H NMR spectrum of compound **1** in chloroform-*d* (600 MHz).

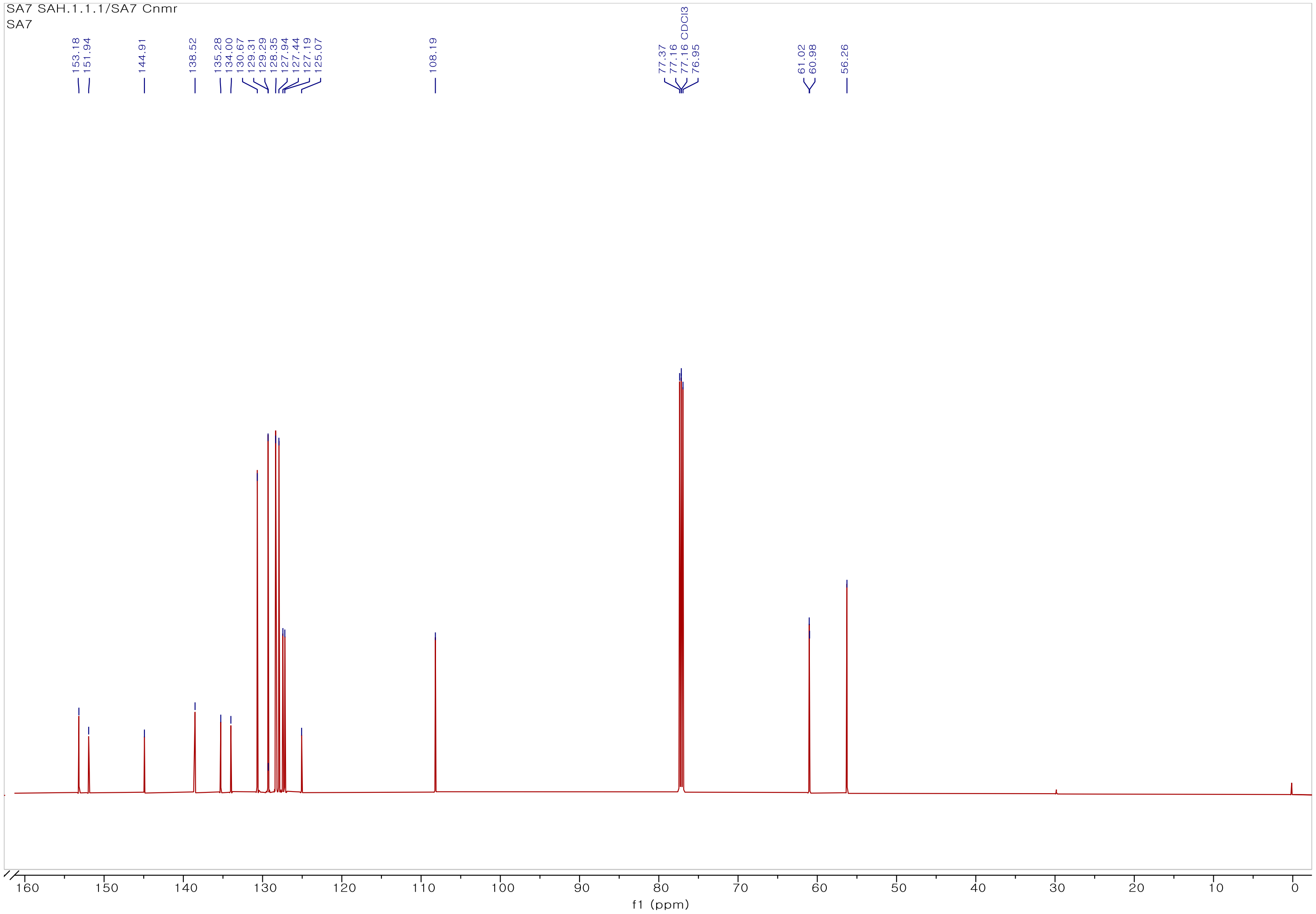

Figure S2. ^{13}C NMR spectrum of compound **1** in chloroform-*d* (150 MHz).

[Mass Spectrum]
Data : EI-B026 Date : 06-Sep-2021 13:42
RT : 1.32 min Scan# : (73,103)
Elements : C 100/0, H 100/0, O 10/0
Mass Tolerance : 10ppm, 5mmu if m/z < 500, 10mmu if m/z > 1000
Unsaturation (U.S.) : -0.5 - 20.0

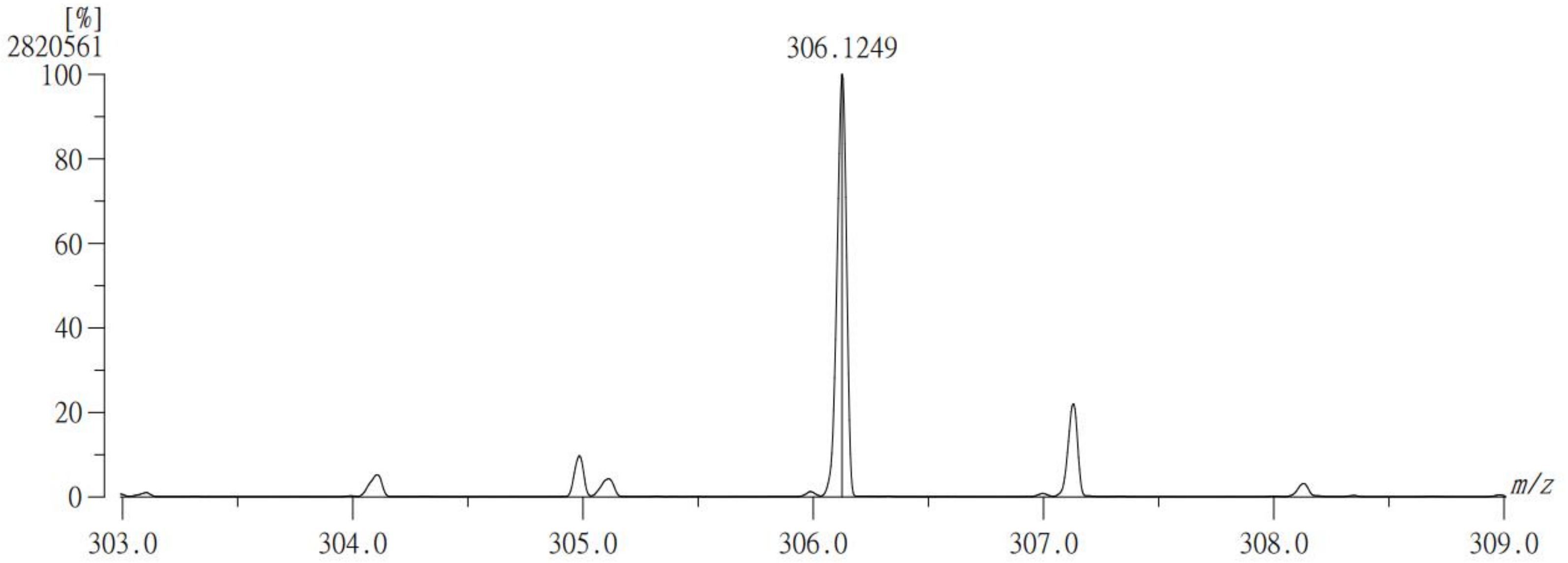

	Observed m/z	Int%	Err[ppm / mmu]	U.S.	Composition
1	306.1249	100.00	-2.3 / -0.7	12.0	C20 H18 O3

Figure S3. HR-EIMS positive spectrum of compound **2.**

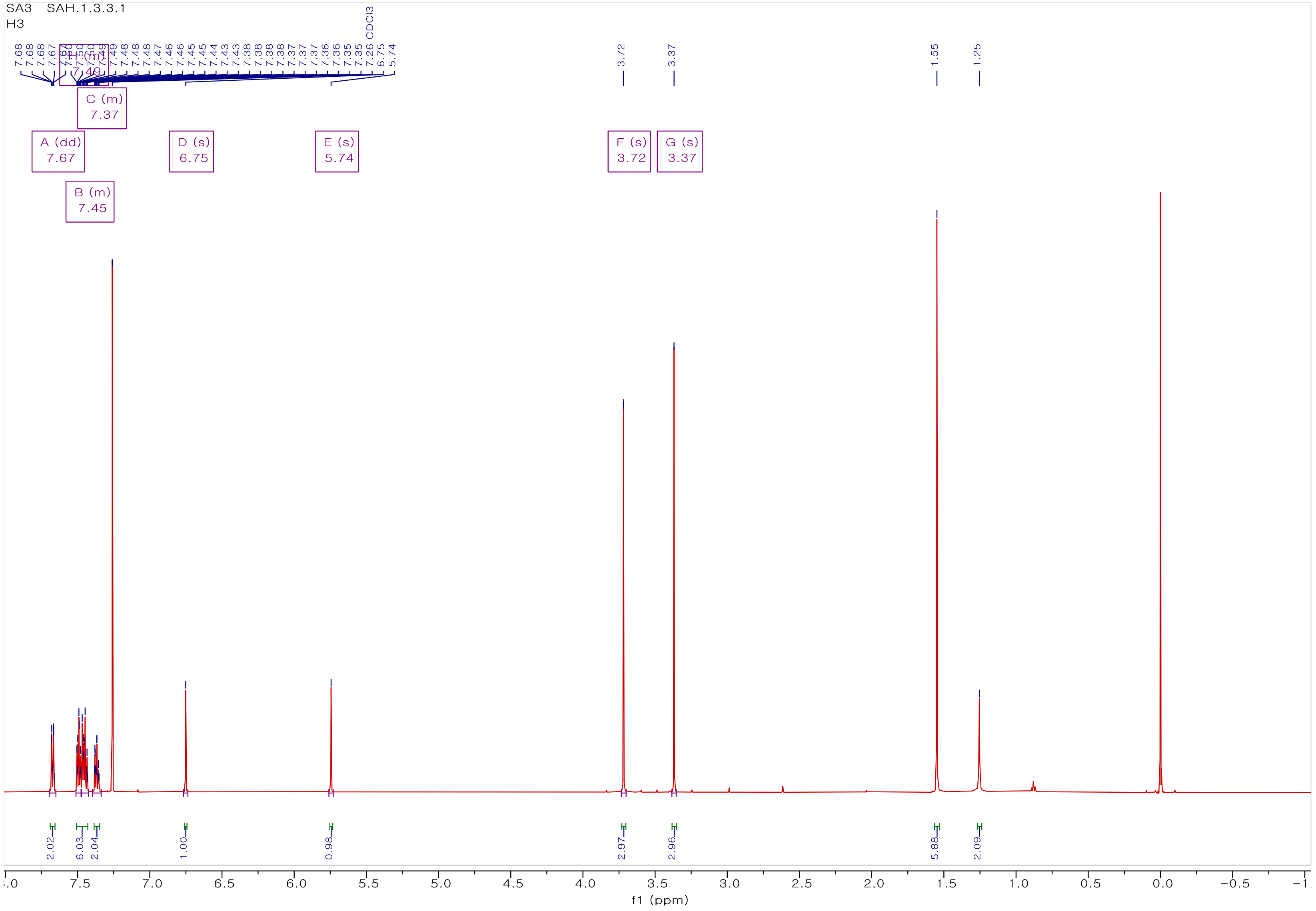

Figure S4. ^{1}H NMR spectrum of compound **2** in chloroform-*d* (600 MHz).

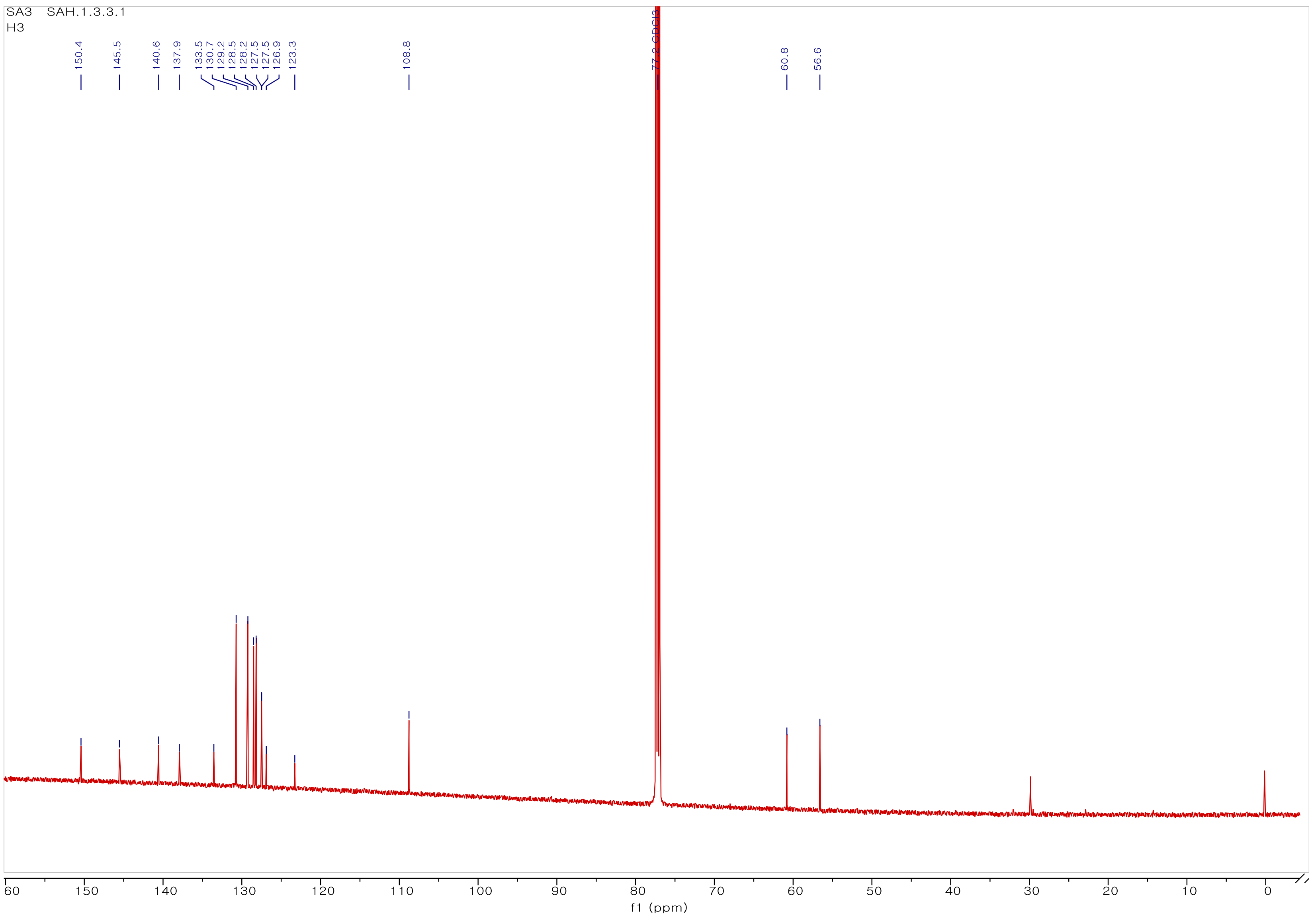

Figure S5. ^{13}C NMR spectrum of compound **2** in chloroform-*d* (150 MHz).

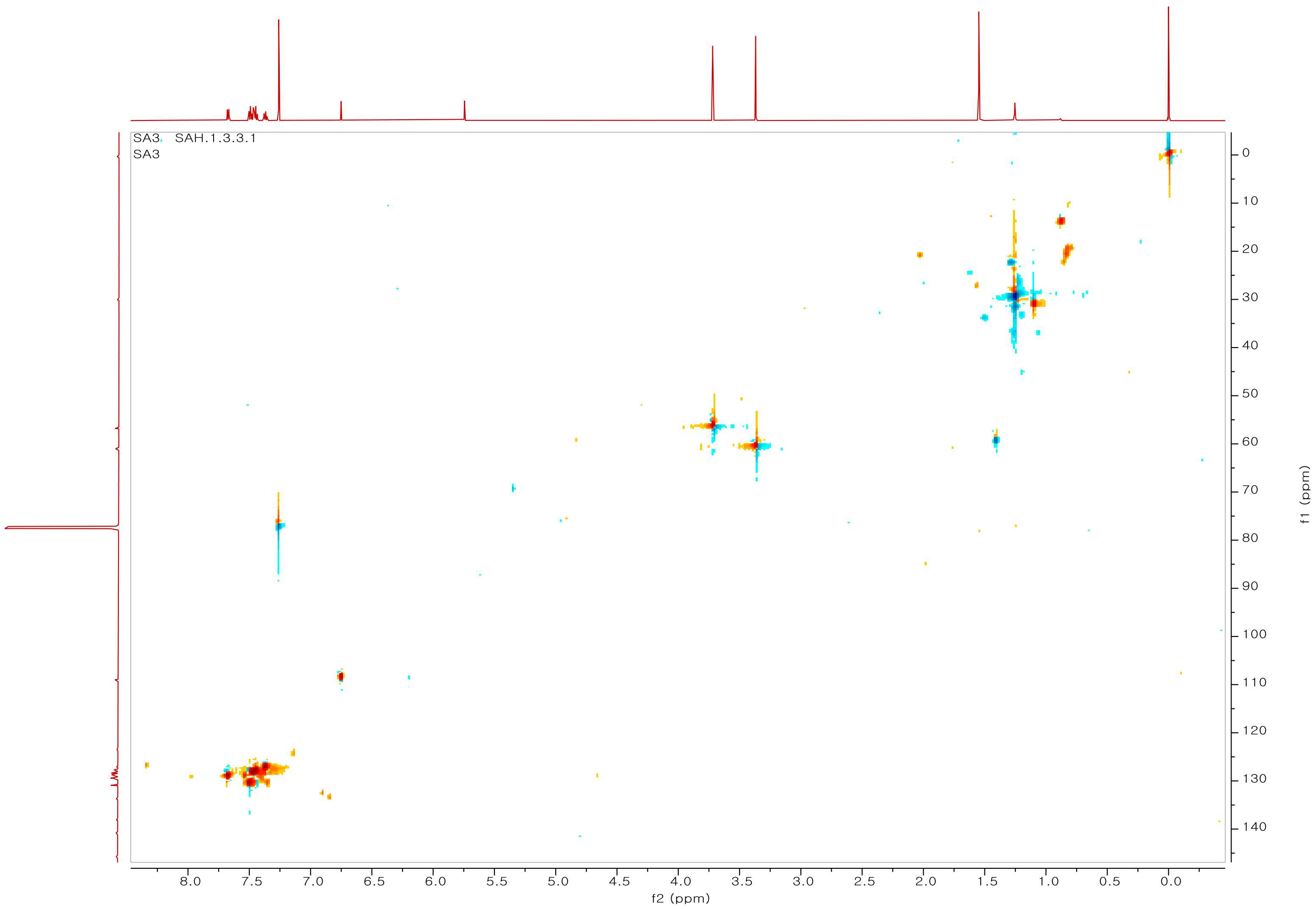

Figure S6. HSQC NMR spectrum of compound **2** in chloroform-*d* (600 MHz).

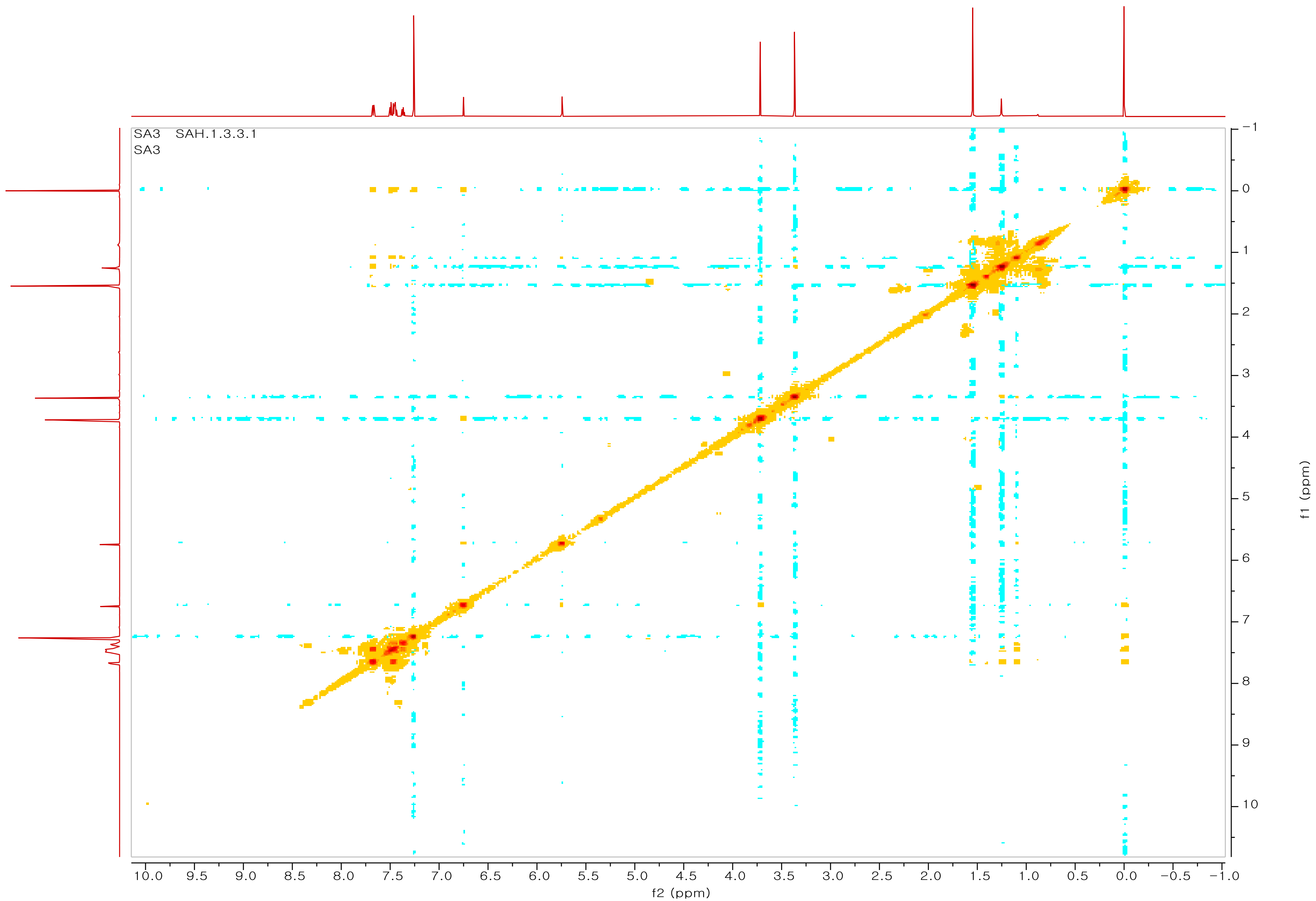

Figure S7. COSY NMR spectrum of compound **2** in chloroform-*d* (600 MHz).

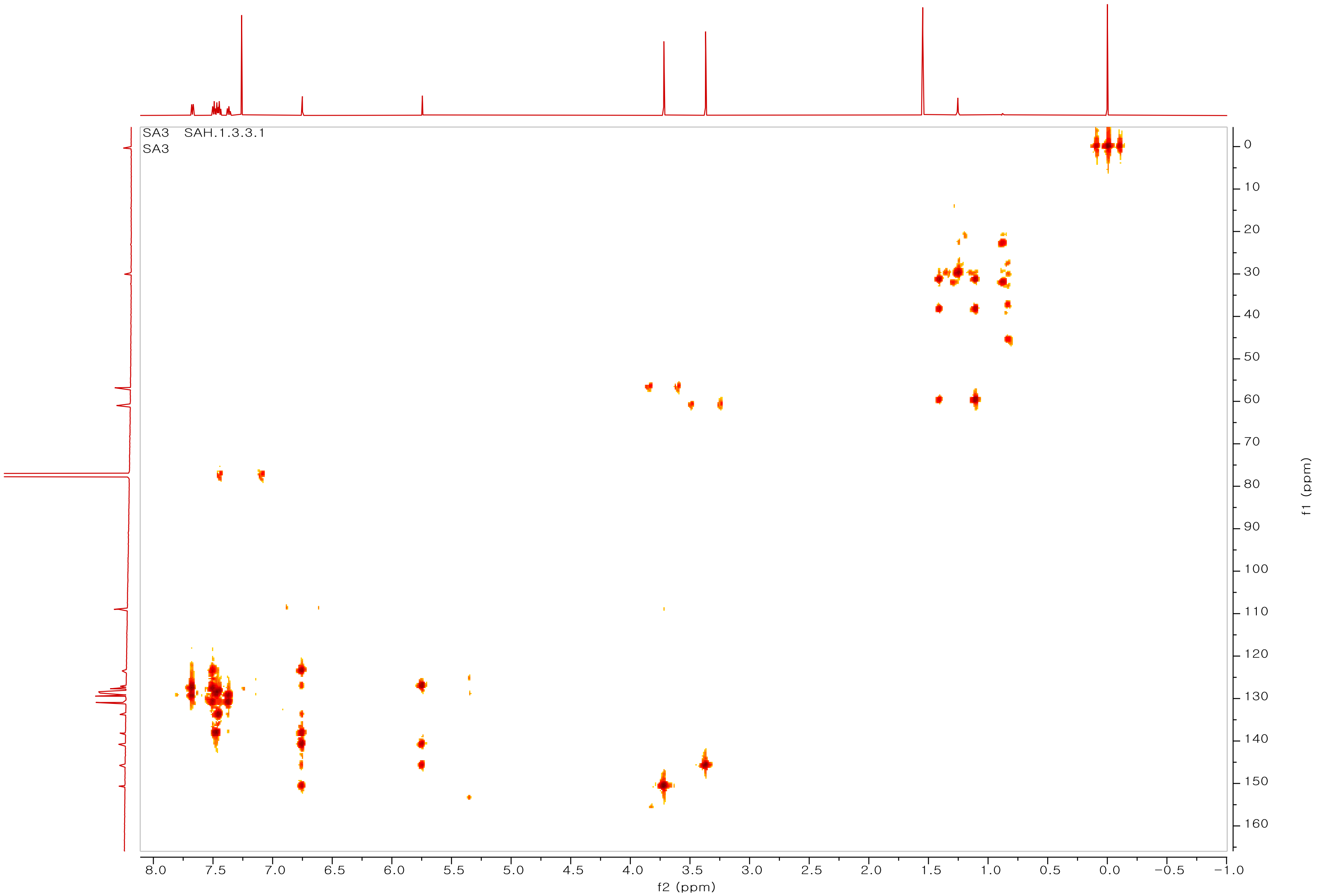

Figure S8. HMBC NMR spectrum of compound **2** in chloroform-*d* (600 MHz).

[Mass Spectrum]
Data : EI-B027 Date : 06-Sep-2021 13:55
RT : 0.88 min Scan# : (49,59)
Elements : C 100/0, H 100/0, O 10/0
Mass Tolerance : 10ppm, 5mmu if m/z < 500, 10mmu if m/z > 1000
Unsaturation (U.S.) : -0.5 - 20.0

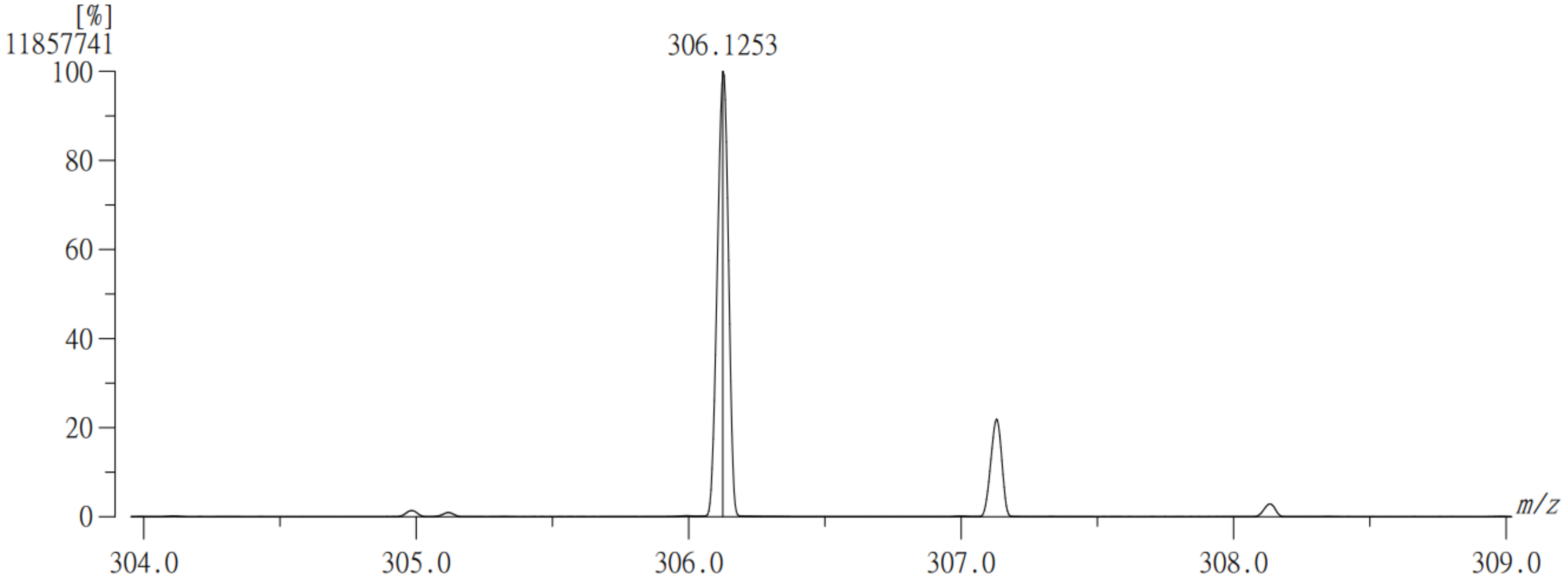

	Observed m/z	Int%	Err[ppm / mmu]	U.S.	Composition
1	306.1253	100.00	-1.0 / -0.3	12.0	C20 H18 O3

Figure S9. HR-EIMS positive spectrum of compound **3.**

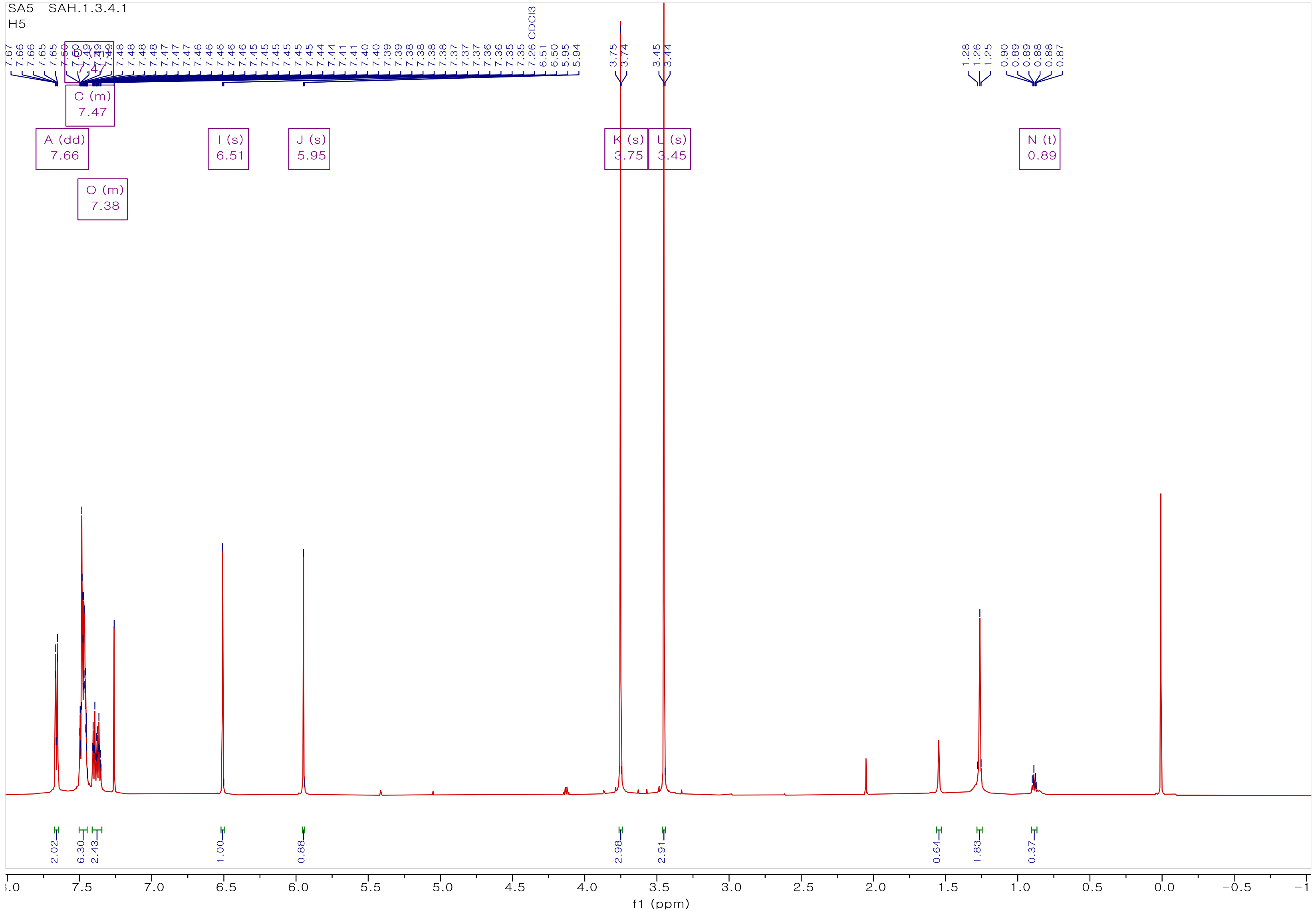

Figure S10. ^{1}H NMR spectrum of compound **3** in chloroform-*d* (600 MHz).

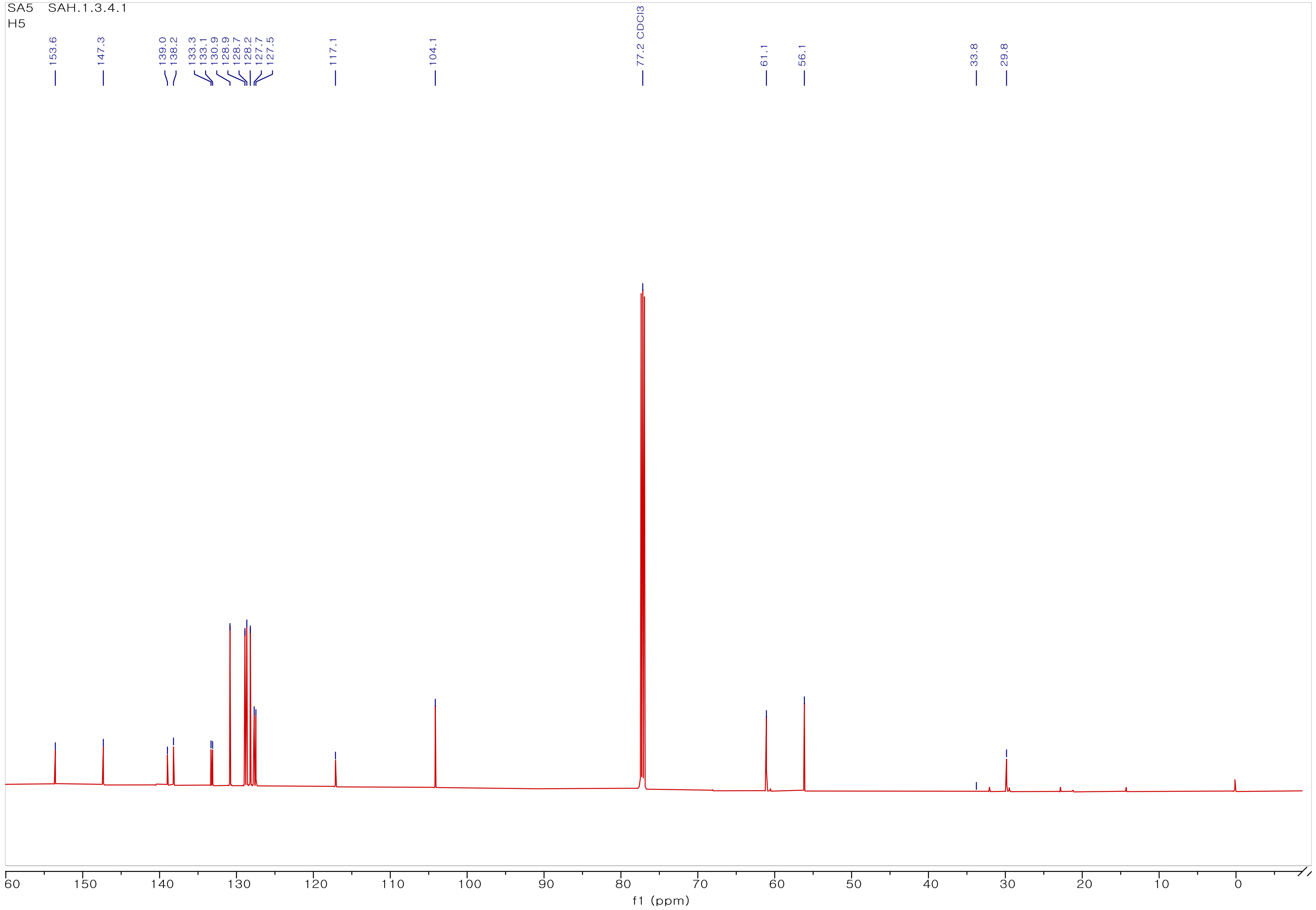

Figure S11. ^{13}C NMR spectrum of compound **3** in chloroform-*d* (150 MHz).

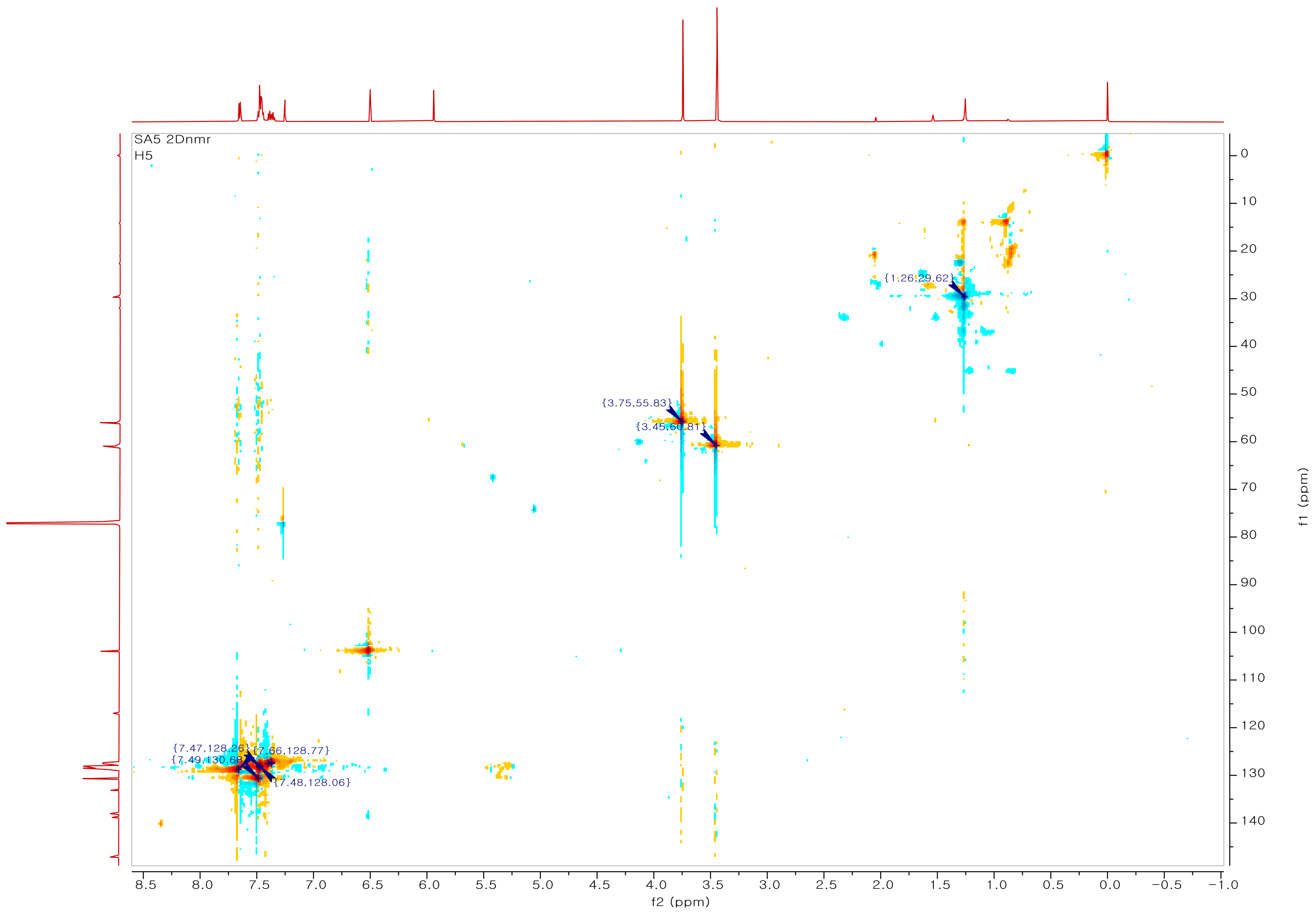

Figure S12. HSQC NMR spectrum of compound **3** in chloroform-*d* (600 MHz).

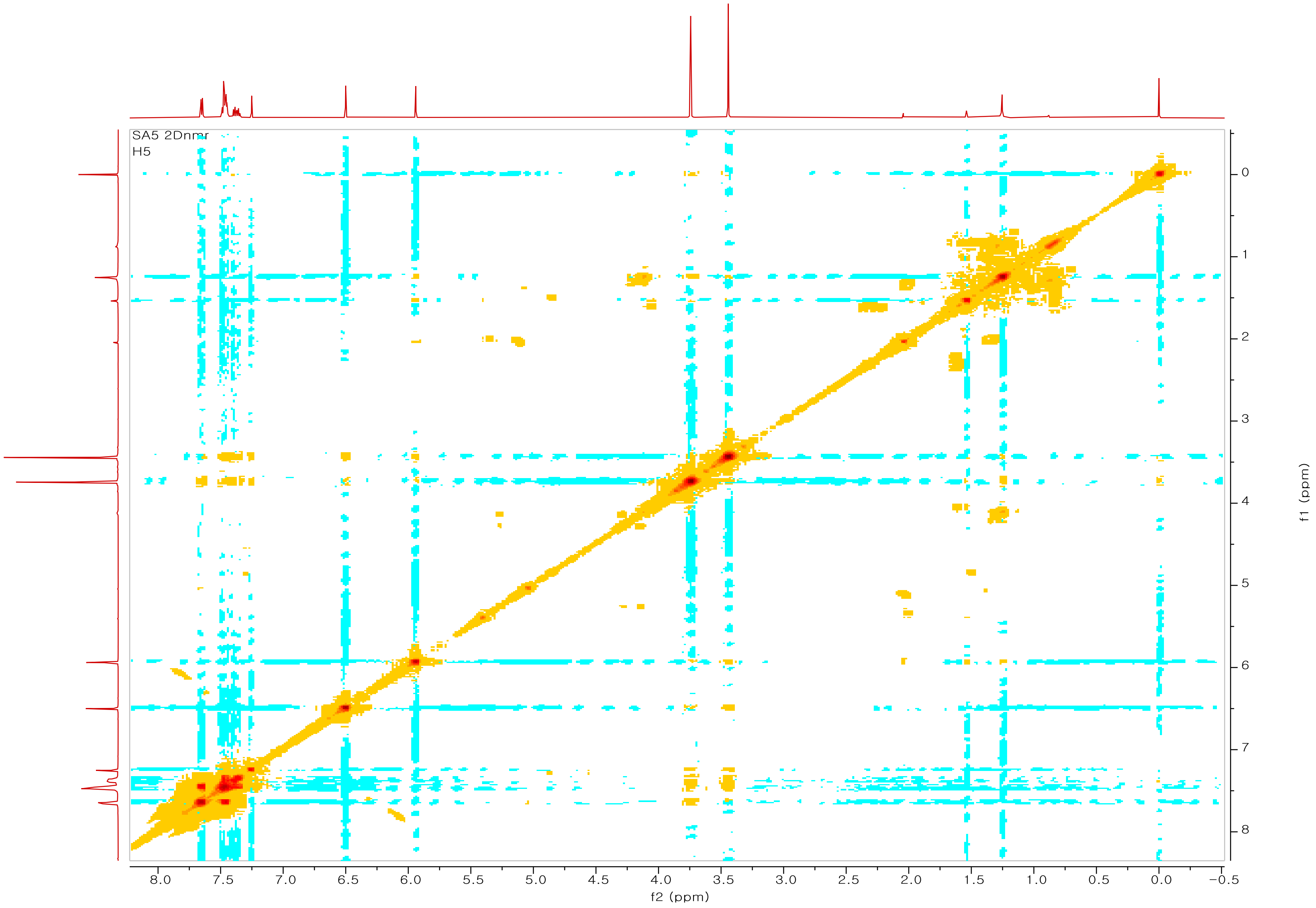

Figure S13. COSY NMR spectrum of compound **3** in chloroform-*d* (600 MHz).

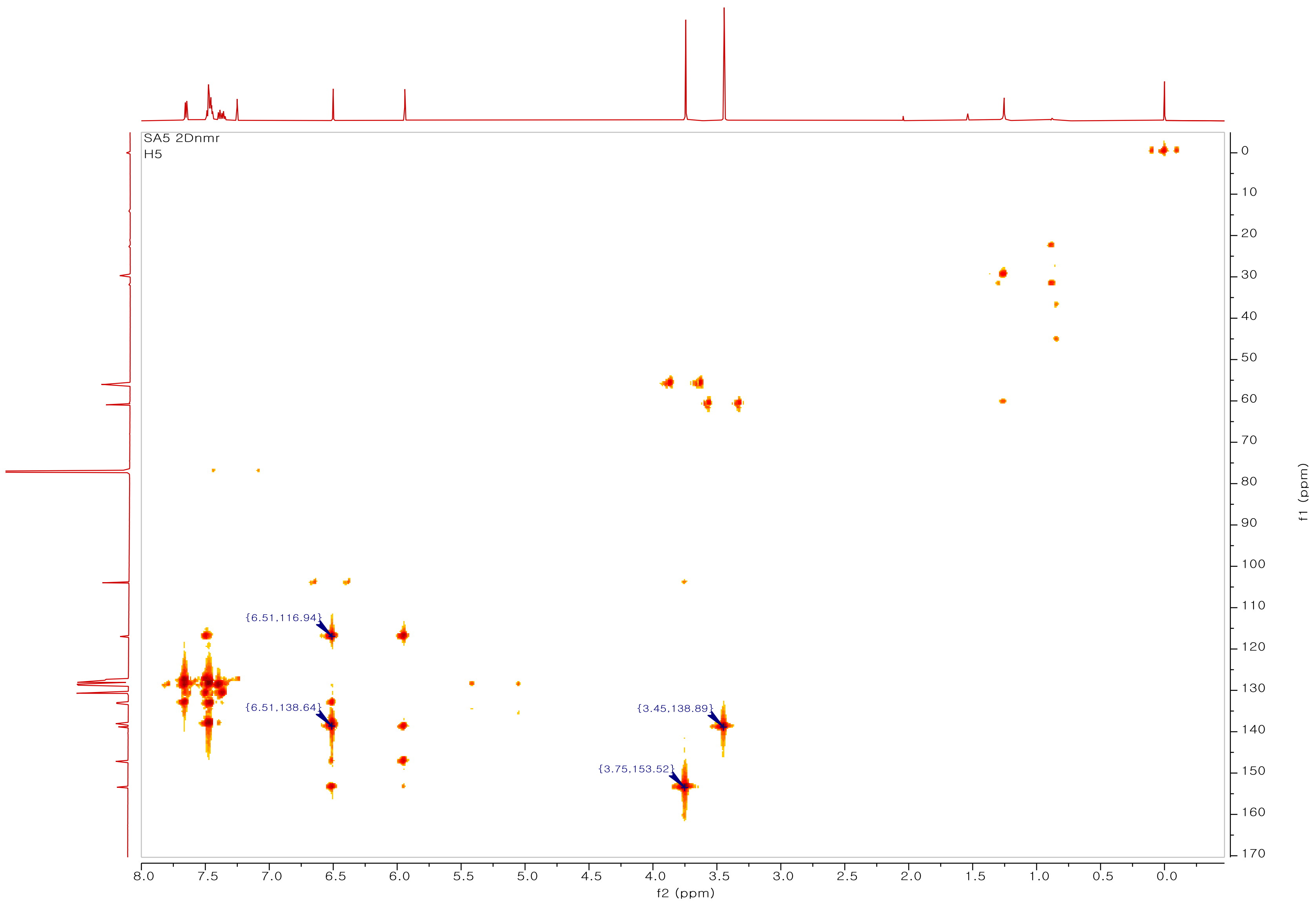

Figure S14. HMBC NMR spectrum of compound **3** in chloroform-*d* (600 MHz).

[Mass Spectrum]
Data : EI-B029 Date : 06-Sep-2021 14:08
RT : 0.74 min Scan# : (42,48)
Elements : C 100/0, H 100/0, O 10/0
Mass Tolerance : 10ppm, 5mmu if m/z < 500, 10mmu if m/z > 1000
Unsaturation (U.S.) : -0.5 - 20.0

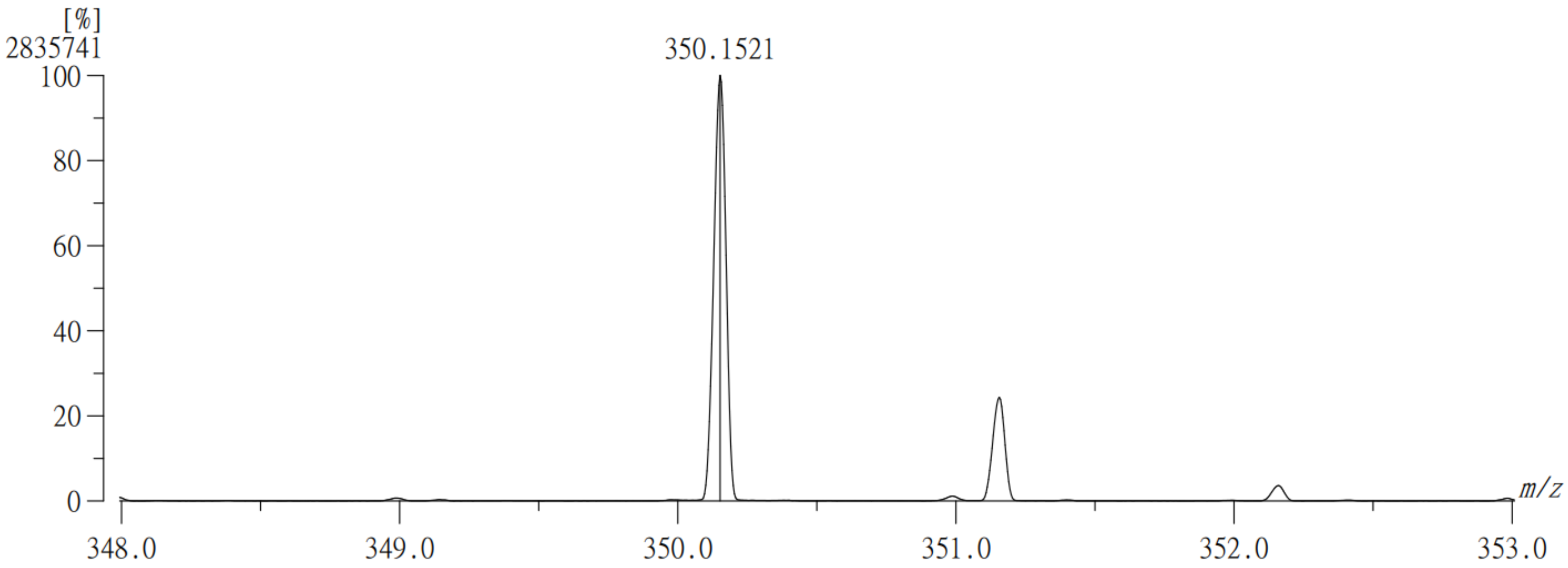

	Observed m/z	Int%	Err[ppm / mmu]	U.S.	Composition
1	350.1521	100.00	+0.8 / +0.3	12.0	C22 H22 O4

Figure S15. HR-EIMS positive spectrum of compound **4.**

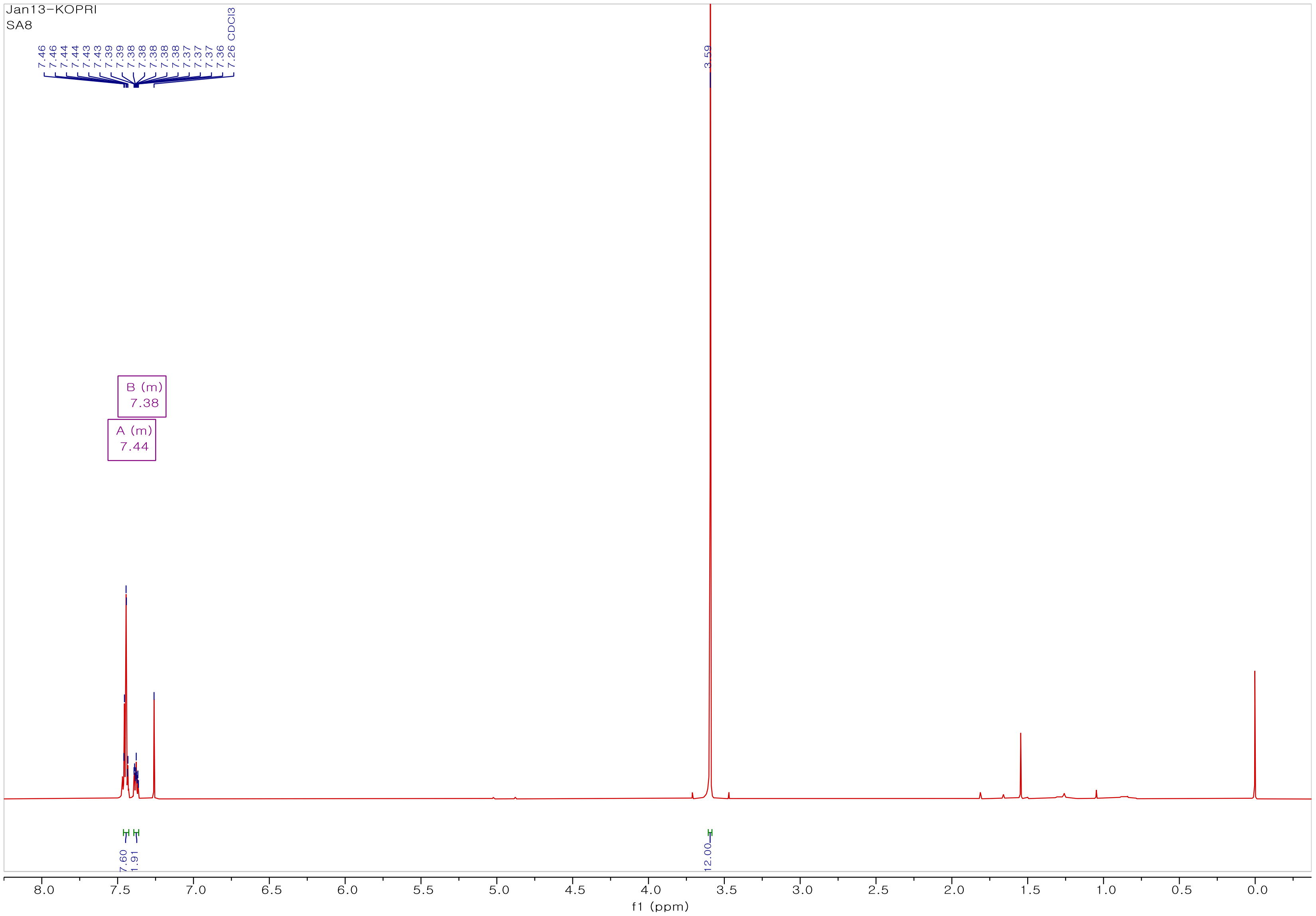

Figure S16. ^{1}H NMR spectrum of compound **4** in chloroform-*d* (600 MHz).

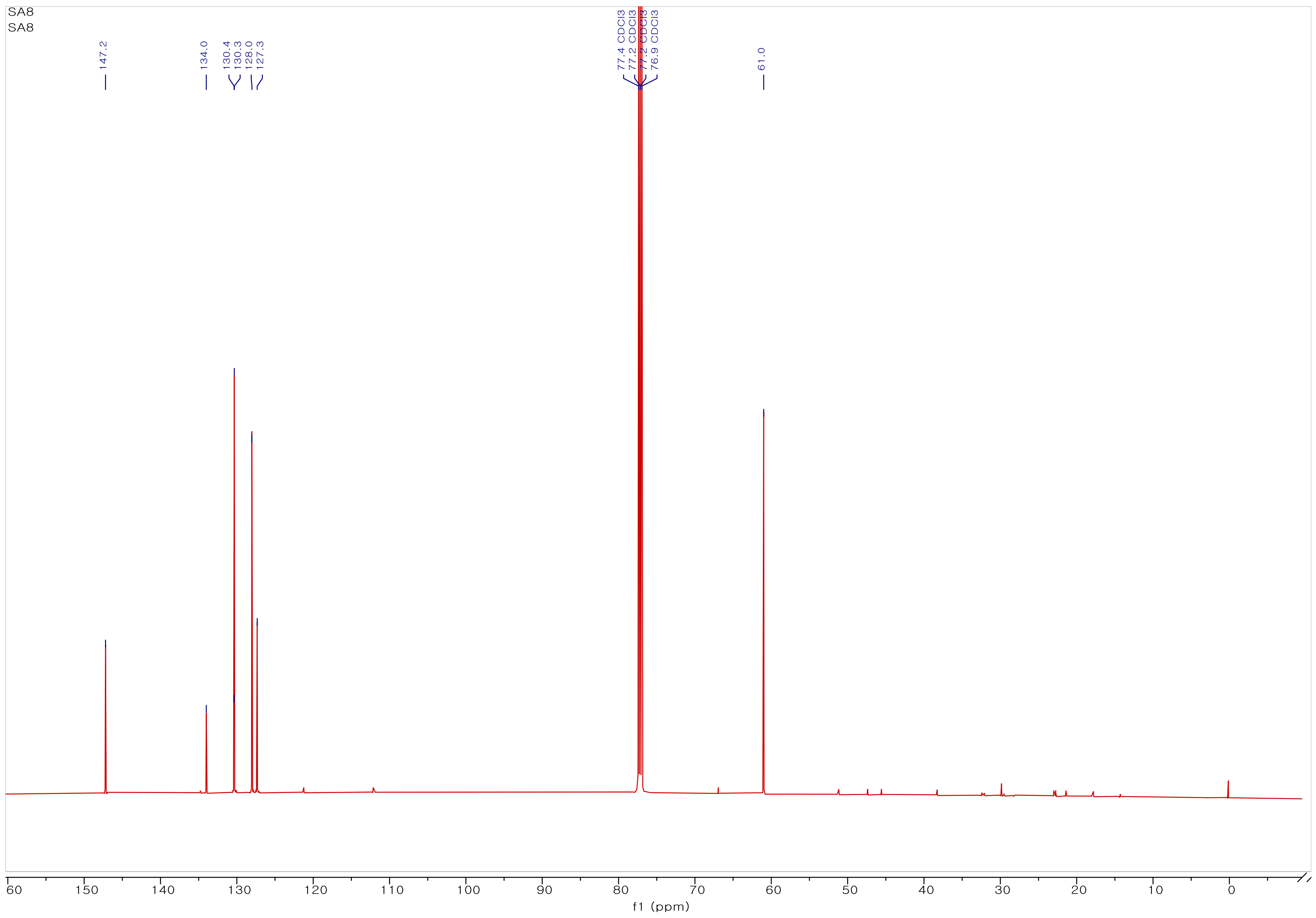

Figure S17. ^{13}C NMR spectrum of compound **4** in chloroform-*d* (150 MHz).

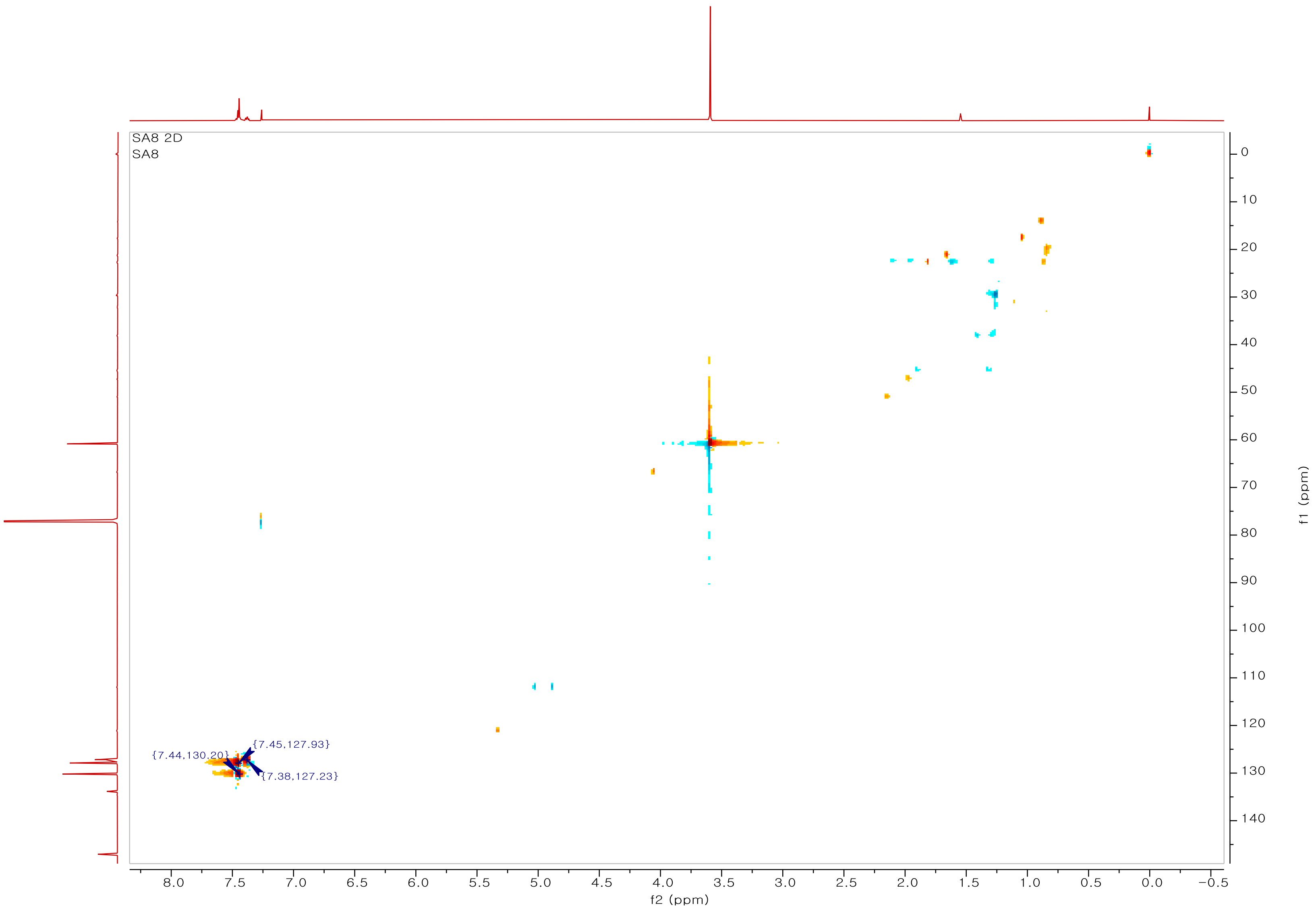

Figure S18. HSQC NMR spectrum of compound **4** in chloroform-*d* (600 MHz).

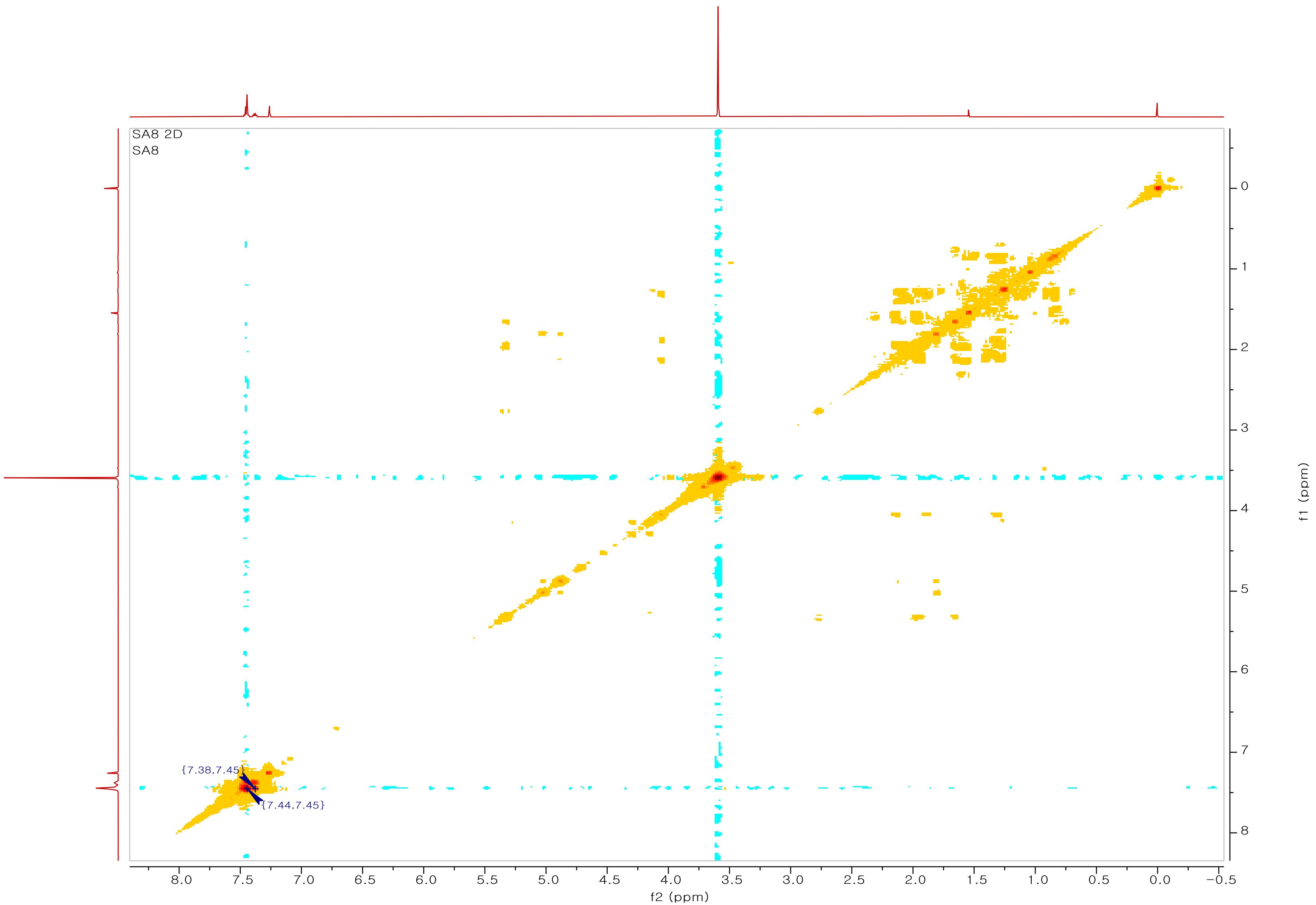

Figure S19. COSY NMR spectrum of compound **4** in chloroform-*d* (600 MHz).

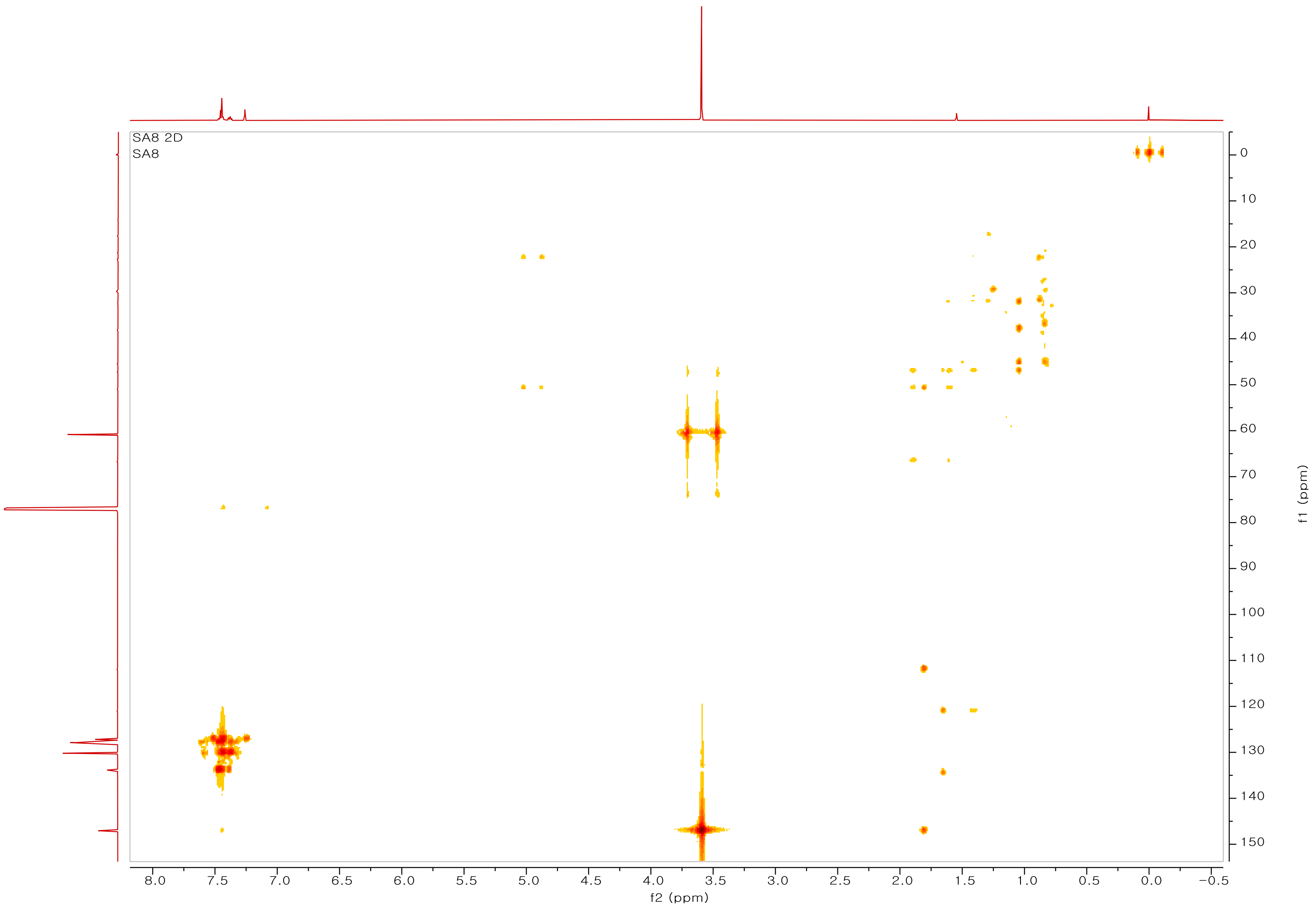

Figure S20. HMBC NMR spectrum of compound **4** in chloroform-*d* (600 MHz).

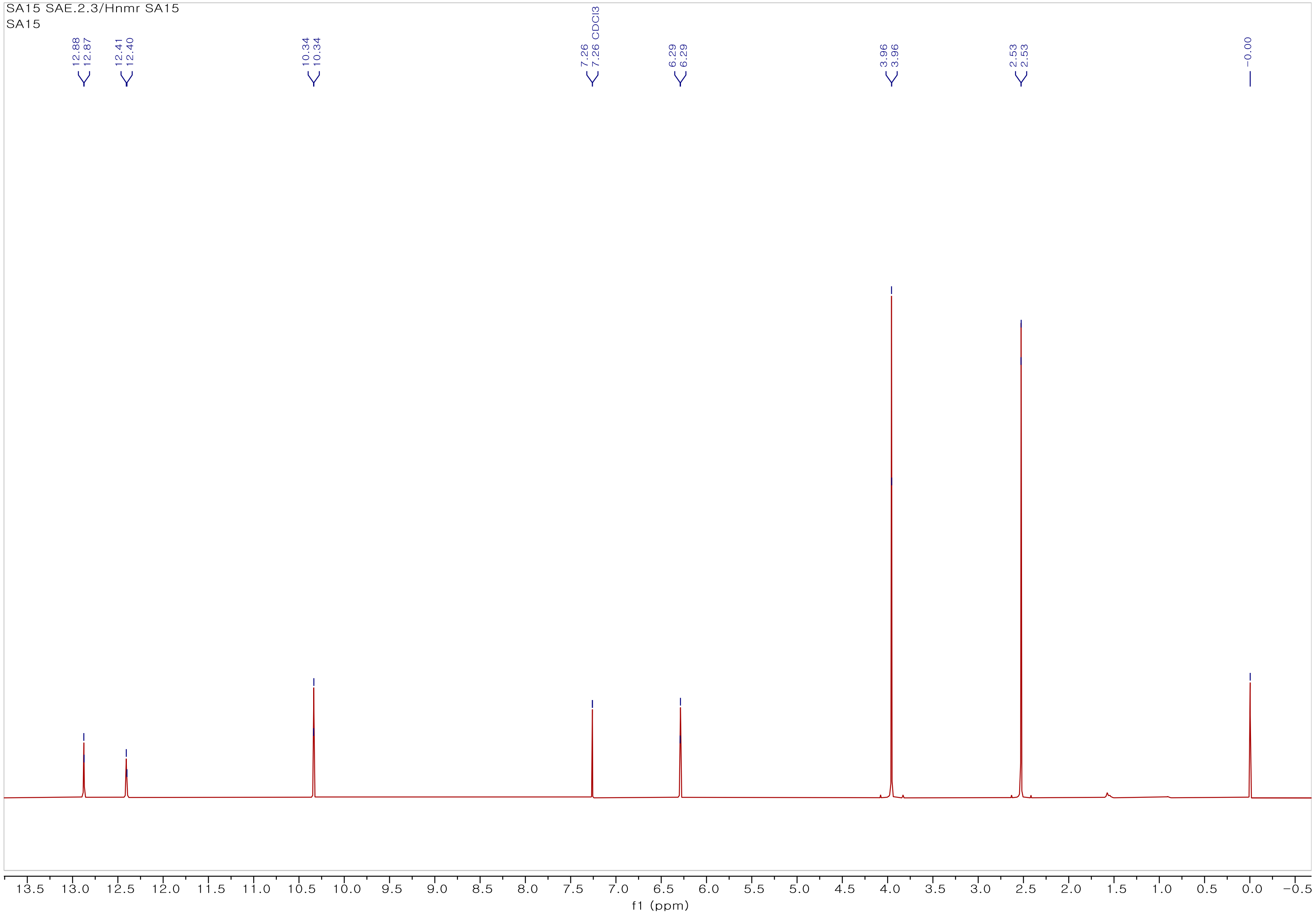

Figure S21. ^{1}H NMR spectrum of compound **5** in chloroform-*d* (600 MHz).

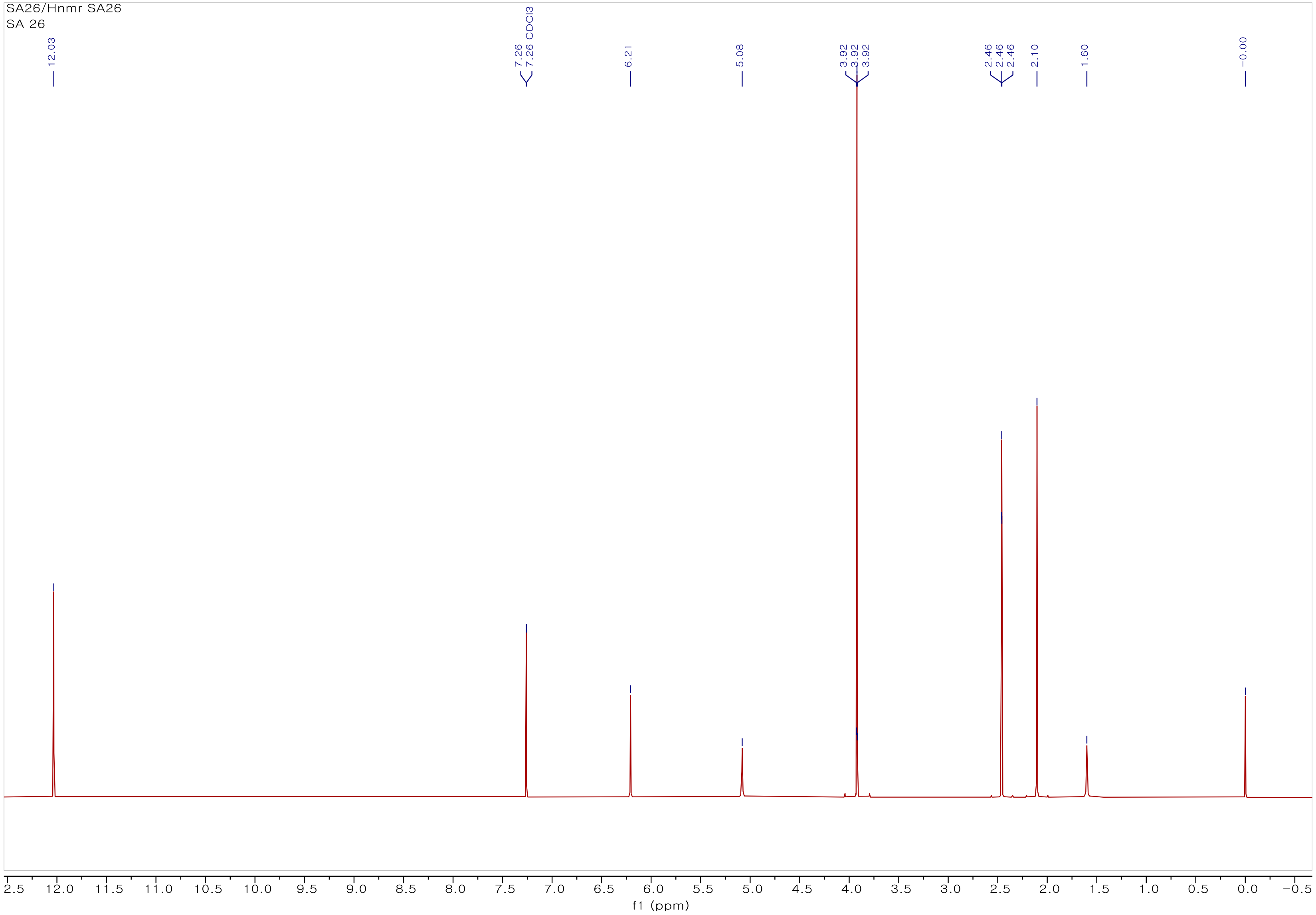

Figure S22. ^{1}H NMR spectrum of compound **6** in chloroform-*d* (600 MHz).

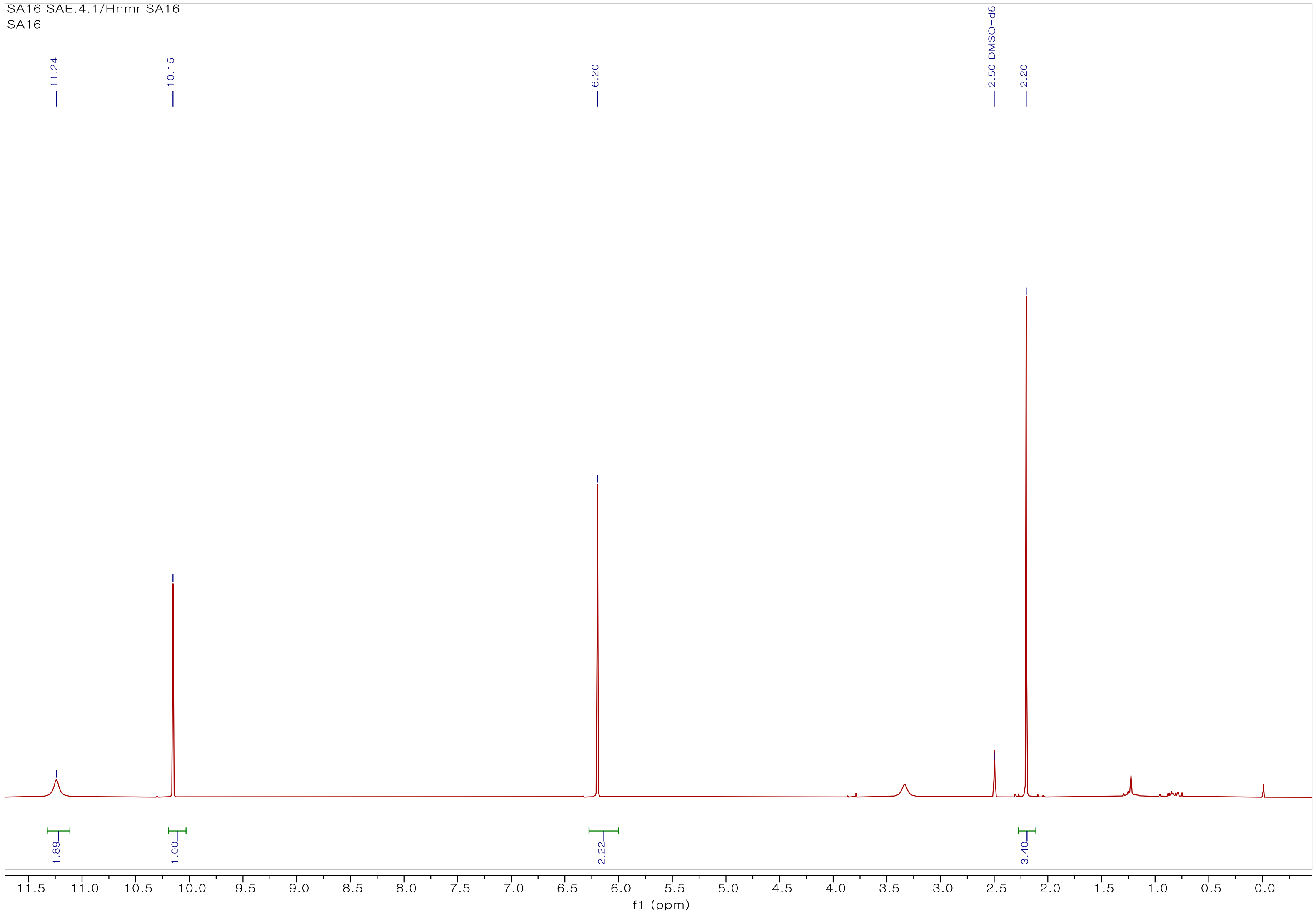

Figure S23. ^{1}H NMR spectrum of compound **7** in DMSO-*d6* (600 MHz).

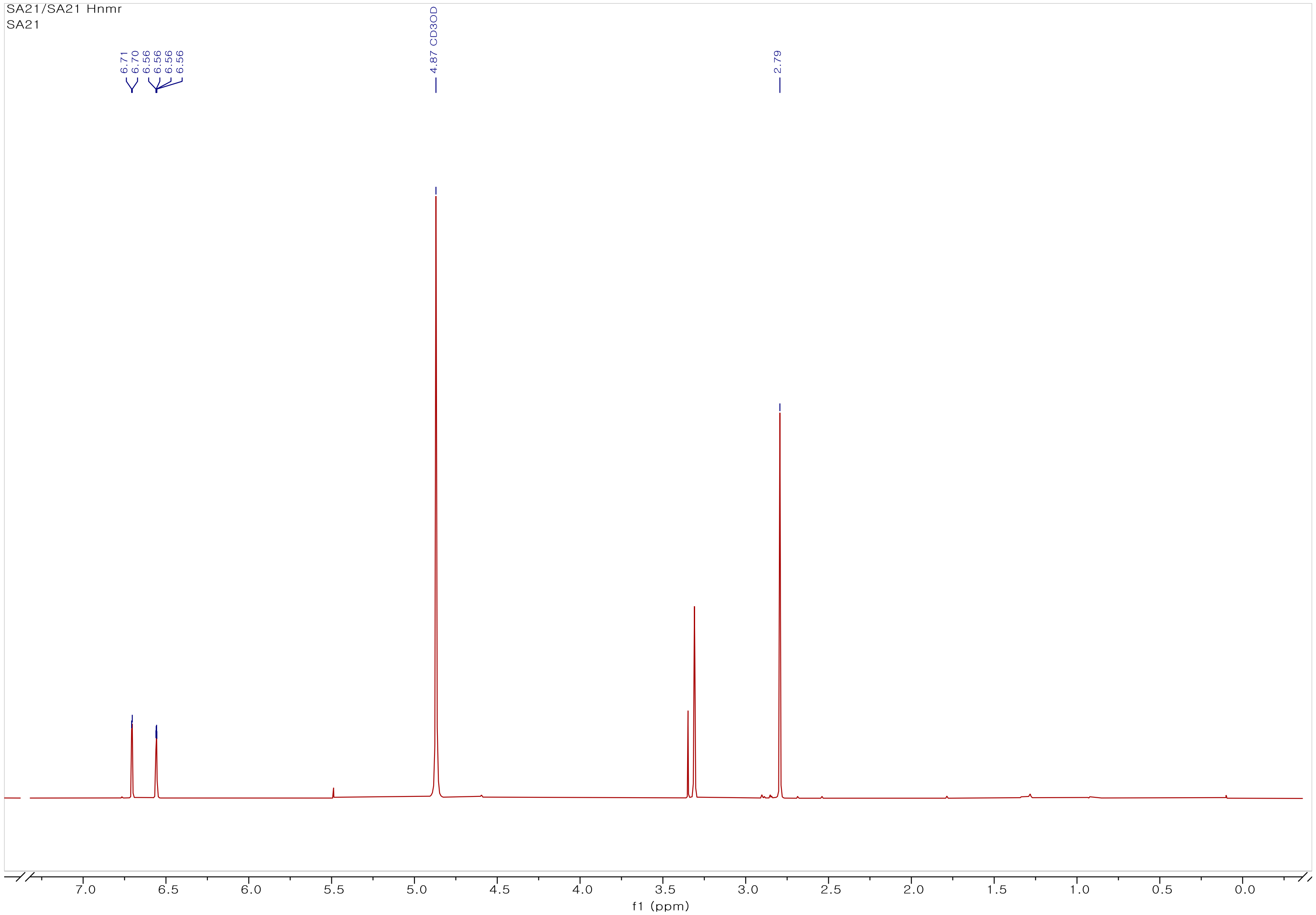

Figure S24. ^{1}H NMR spectrum of compound **8** in methanol-*d4* (600 MHz).

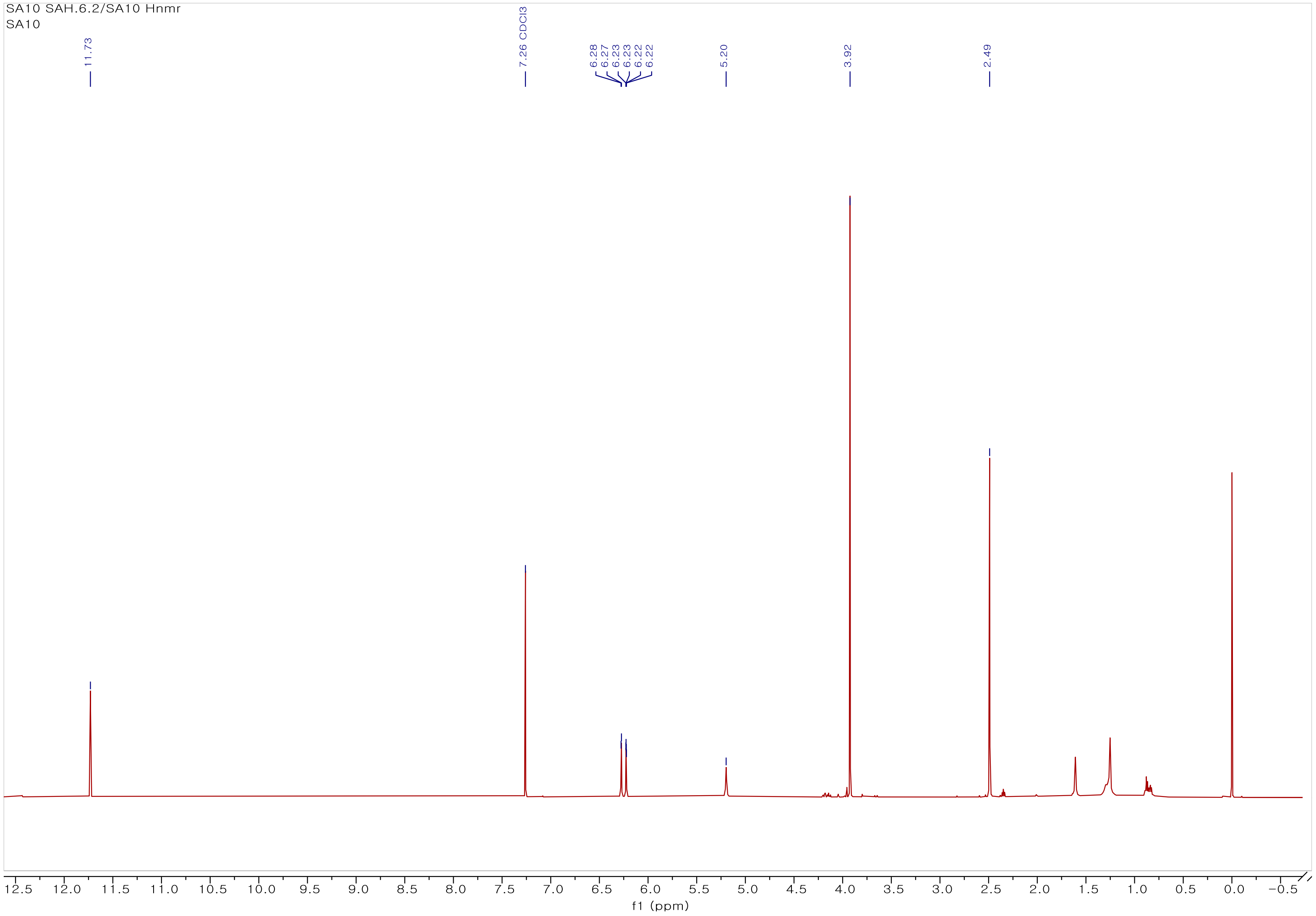

Figure S25. ^{1}H NMR spectrum of compound **9** in chloroform-*d* (600 MHz).

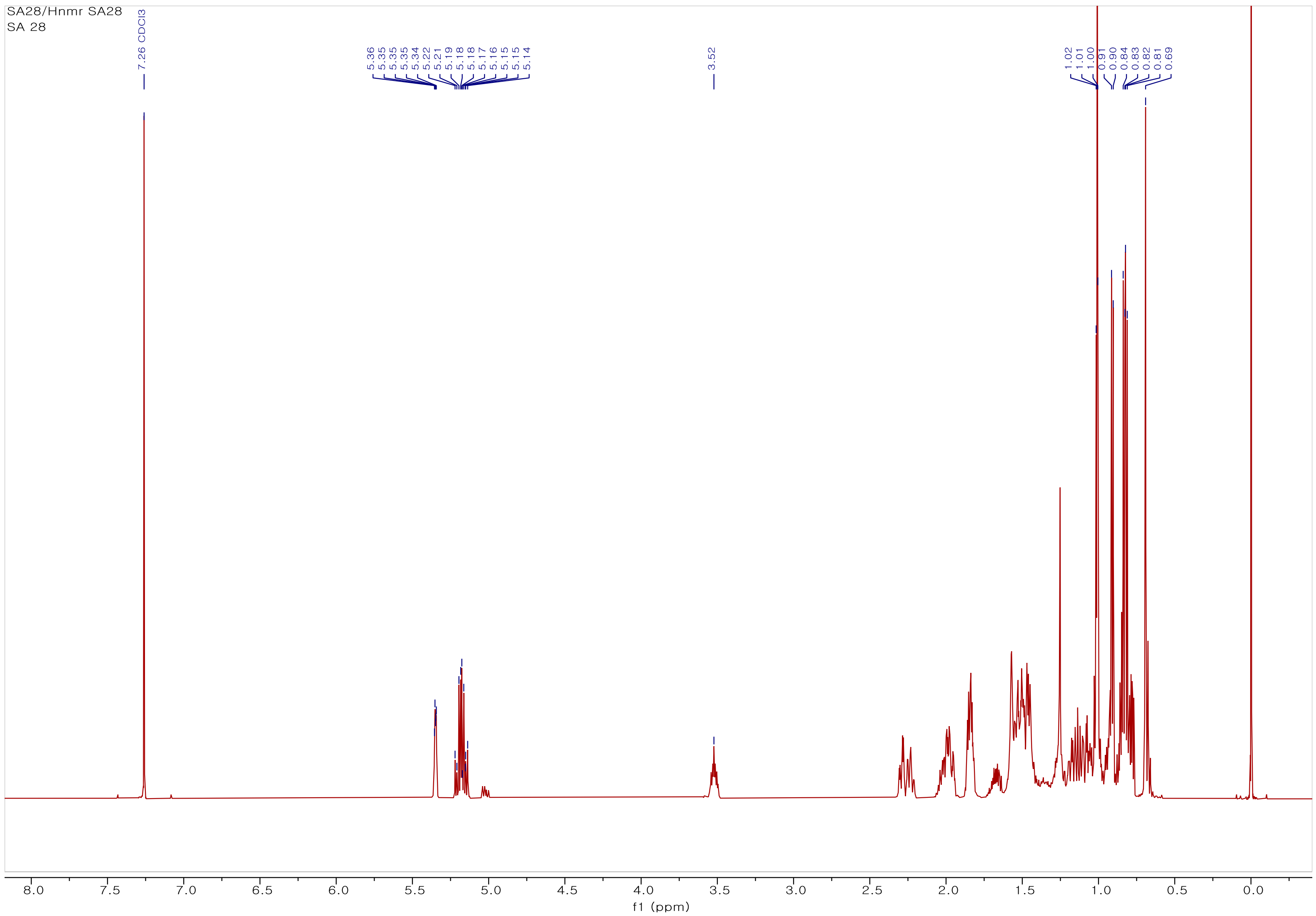

Figure S26. ^{1}H NMR spectrum of compound **10** in chloroform-*d* (600 MHz).

Table S1. Cytotoxicity of compounds **3** against HCT116 cells.

Compound	IC_{50} (μM)[a]
**3**	3.76 ± 0.03

[a]IC50 value of each compound was defined as the concentration (μM) that caused 50% inhibition of NO production in LPS-activated RAW264.7 mouse macrophages. The results are the averages of three independent experiments, and the data are expressed as mean ± SD.

Table S2. NO inhibition of compounds **3** against LPS-induced RAW264.7 cells.

Compound	IC_{50} (μM)[a]
**3**	22.82 ± 0.015

[a]IC_{50} value of each compound was defined as the concentration (μM) that caused 50% inhibition of NO production in LPS-activated RAW264.7 mouse macrophages. The results are the averages of three independent experiments, and the data are expressed as mean ± SD.
